# Comparative effectiveness of intra-articular therapies in knee osteoarthritis: a meta-analysis comparing platelet-rich plasma (PRP) with other treatment modalities

**DOI:** 10.1097/MS9.0000000000001615

**Published:** 2023-12-15

**Authors:** Saad Khalid, Abraish Ali, FNU Deepak, Muhammad Sibtain Zulfiqar, Laiba Urooj Malik, Zubaida Fouzan, Rabiya Ali Nasr, Maryam Qamar, Pratik Bhattarai

**Affiliations:** a Department of Medicine, Dow University of Health Sciences; b Shaheed Mohtarma Benazir Bhutto Medical College Lyari; c Dow International Medical College, Karachi; d Services Institute of Medical Sciences; e King Edward Medical University, Lahore; f Rawalpindi Medical University, Rawalpindi, Pakistan; g Manipal College of medical Sciences, Pokhara, Nepal

**Keywords:** corticosteroids, hyaluronic acid, knee osteoarthritis, meta-analysis, platelet-rich plasma

## Abstract

**Introduction:**

Knee osteoarthritis (KOA) is a progressive joint disease commonly treated with intra-articular injections, including platelet-rich plasma (PRP), hyaluronic acid (HA), or corticosteroids (CS). This updated meta-analysis aims to enhance the statistical power of the results and provide comprehensive clinical evidence that reflects the most current research. By doing so, the authors aim to suggest a reliable estimate for the development of guidelines, addressing the pressing need for effective and minimally invasive treatment options.

**Methods:**

PubMed, Scopus, clinicaltrials.gov, Cochrane Central were searched until March 2023, for randomized controlled trials (RCTs) comparing the effectiveness of intra-articular injectable therapies, including PRP, HA, CS, and placebo, in KOA. Data extraction involved baseline characteristics and outcome measures [Western Ontario and McMaster Universities Arthritis Index (WOMAC) scores, Visual Analog Scale (VAS) pain scores, KOOS, and IKDC scores] at 1, 3, 6 and 12 months. Statistical analysis, including subgroup analysis, assessment of heterogeneity, and publication bias, was conducted using Review Manager.

**Results:**

Our meta-analysis of 42 studies involving 3696 patients demonstrated that PRP treatment resulted in significant pain relief compared to HA injections, as evidenced by improved WOMAC pain (MD: −0.74; 95% CI: −1.02 to −0.46; *P*≤0.00001; *I* ^2^=94%) and VAS pain (MD: −0.65; 95% CI: −1.24 to −0.06; *P*=0.03; I^2^=97%) outcomes. Similarly, PRP showed greater efficacy in reducing WOMAC pain (MD: −8.06; 95% CI: −13.62 to −2.51: *P*=0.004; *I* ^2^=96%) and VAS pain (MD: −1.11; 95% CI: −1.64 to −0.59; *P*≤0.0001; *I* ^2^=68%) compared to CS injections, with the most significant improvement observed at 6 months.

**Conclusions:**

PRP is an effective treatment for KOA. It provides symptomatic relief, has the potential to reduce disease progression, and has sustained effects up to 12 months. PRP offers superior pain relief and functional enhancement compared to CS and HA injections.

## Introduction

Highlights

- Knee osteoarthritis is a widespread condition causing pain and reduced quality of life.
- Various treatments option exist, including platelet-rich plasma (PRP) therapy, but current guidelines lack clear recommendations due to limited evidence.
- Our meta-analysis found that PRP treatment significantly reduced pain compared to hyaluronic acid (HA) and corticosteroid (CS) injections, as demonstrated by improved Western Ontario and McMaster Universities Arthritis Index and Visual Analog Scale pain scores.
- The most significant improvement observed at 6 months.
- PRP effectively treats knee osteoarthritis, providing lasting pain relief, potential disease progression reduction, and superior results compared to CS and HA injections.

Osteoarthritis (OA) is a degenerative joint disease involving all joints, while knee osteoarthritis (KOA) is a multi-morbid disability of the knee joint characterized by knee pain, inflammation, and articular degeneration that leads to not only an increase in health care burden but also has a major effect on an individual’s quality of life^1^. Advancing age^2^, female sex^3^, obesity, inflammation^4,5^ and lower adherence to the Mediterranean diet^6^ are risk factors in progression of KOA^2–5^. PRP is an autologous blood derivative with high growth factors such as transforming growth factor, platelet-derived growth factor, insulin-like growth factor, vascular endothelial growth factor, vascular endothelial growth factor and bioactive proteins, affecting the healing of bone, cartilage, ligament and tendon^7^. Therapies are evolving in markets such as hyaluronic acid (HA), platelet-rich plasma (PRP), ozone gas, saline, corticosteroids (CS) and mesenchymal stem cell therapy^8,9^. HA and intra-articular CS play an anti-inflammatory role in KOA and release pain and inflammation^10,11^. Recent research has also focused on using mesenchymal stem cells (MSCs), derived from sources such as adipose tissue, bone marrow and umbilical cord blood, for treating OA. MSCs show promise in slowing cartilage degradation in OA by regulating the immune response and releasing beneficial compounds^9^. In addition, according to recent evidence, PRP therapy reduces pain and stiffness and delays articular degeneration in patients with mild to moderate KOA^12^. To enhance the quality of life in patients with KOA, it is necessary to compare the effects of various therapies with PRP.

Despite an increasing body of literature on the effectiveness of PRP in mild to moderate KOA, current guidelines from the American Academy of Orthopedic Surgeons (AAOS) do not provide a clear recommendation for or against its use due to insufficient scientific evidence^13^. Additionally, a recent position paper by the American Association of Hip And Knee Surgeons (AAHKS) also does not recommend PRP for advanced hip and knee arthritis due to insufficient evidence regarding its efficacy^14^. Although a recent network meta-analysis suggests that platelet-rich plasma therapy may be as effective as or more effective than other intra-articular therapies, the authors were unable to make clinical recommendations for PRP use in KOA due to methodological flaws and limitations in the included studies^15^. Therefore, we conducted an updated systematic review and meta-analysis, incorporating recently published trials, to increase statistical power and strengthen clinical evidence on the efficacy of PRP compared to other intra-articular therapies for KOA. The findings of this analysis can contribute to the formulation of clinical guidelines for the treatment of KOA.

## Methods

This meta-analysis conforms to the Preferred Reporting Items for Systematic Reviews and Meta-analyses (PRISMA) 2020 recommendations, Supplemental Digital Content 1, http://links.lww.com/MS9/A332 ^16^. The protocol of this systematic review and meta-analysis was registered on PROSPERO.

### Literature search and study selection

We conducted an extensive electronic literature search on PubMed/MEDLINE, Scopus, clinicaltrials.gov, Cochrane Central (in the Cochrane Library) and Google Scholar from inception until March 2023 to identify studies that compared the effectiveness of intra-articular injectable therapies with PRP. For literature search, following keywords and MeSH term combinations were used:

(Platelet-rich plasma OR platelet-rich growth factors OR platelet-rich fibrin OR platelet concentrates) AND (Interarticular corticosteroid injection OR Triamcinolone injection OR corticosteroid shots OR corticosteroids OR steroids OR hyaluronic acid OR Sodium Hyaluronate OR Vitrax OR billon OR Etamucine OR hyvisc OR Luronit OR Amvisc OR healing OR placebo) AND (Knee osteoarthritis OR patellofemoral arthritis OR kneecap arthritis OR degenerative joint disease OR wear and tear arthritis of knee OR osteoarthritis of the knee) were used.

After the initial search, duplicates were removed, and abstracts were then screened independently by two reviewers. This was followed by full-text eligibility screening, also conducted by two independent reviewers. Any discrepancies on study eligibility were resolved by consultation by a third reviewer. Additionally, reference list of included studies was also searched to identify more studies.

### Eligibility criteria

The studies selected were based on a strict eligibility criteria. All Randomized controlled trials (RCTs) comparing the effects of injectable therapies like hyaluronic acid, steroids, placebo, ozone, etc. with PRP on knee osteoarthritis were included. Outcome measures were Western Ontario and McMaster Universities Arthritis Index (WOMAC) scores, Visual Analog Scale (VAS) pain scores, Knee Injury and Osteoarthritis Outcome Score (KOOS), and International Knee Documentation Committee (IKDC). We excluded all types of reviews articles, cross-sectional studies, observational studies, case reports, case series, editorials, commentaries, and animal-based studies, as well as any studies that were not published in the English language. Studies including individuals with recent or imminent knee surgery, or patients who had prosthetic implants, were also eliminated.

### Data extraction

Data was extracted on Microsoft Excel. Baseline characteristics extracted were as follows: Author, year of publication, sample size of study population in each intervention group, mean age, sex, and mean BMI of patients in each group, mean baseline WOMAC scores, mean baseline VAS score. To assess the efficacy of PRP treatment versus other injectables, the outcomes measures that were compared were: the mean WOMAC pain, stiffness, function, and total scores at 1 month, 3 months, 6 moths, and 12 months; mean VAS pain scores at 1, 3, 6, and 12 months; mean IKDC scores at 1, 3, 6, and 12 months and KOOS pain scores at 1, 3, 6, and 12 months. Quality assessment of the studies included was carried out by two reviewers using Cochrane risk of bias tool for Randomized Controlled Trials^17^. In addition, A Measurement Tool to Assess systematic Reviews 2 (AMSTAR 2) checklist was used to self-evaluate this meta-analysis (Supplemental Digital Content 2, http://links.lww.com/MS9/A333)^18^.

### Statistical analysis

Review Manager-v 5.4.1 was used for the statistical analysis. The included studies’ mean differences (MD) were estimated with 95% CIs. To pool the effect sizes across studies, a random effects model was applied. Additionally, we performed a subgroup analysis of different intra-articular treatment modalities including HA, CS and placebo, comparing them with PRP. We hoped to find any changes in treatment effects between different intra-articular therapies by analyzing the subgroups. A *P* value of less than 0.05 was deemed significant. We used the *I* ^*2*^ statistic to examine heterogeneity and considered it significant if *I* ^*2*^ was greater than 75%. To ensure the robustness of our findings, we conducted a leave-one-out sensitivity analysis when high heterogeneity was observed. This analysis involved iteratively removing one study at a time. we performed sensitivity or leave-one-out analysis. In addition, funnel plots and Egger’s test were used to assess publication bias.

## Results

### Literature search and quality assessment

**Figure 1:**
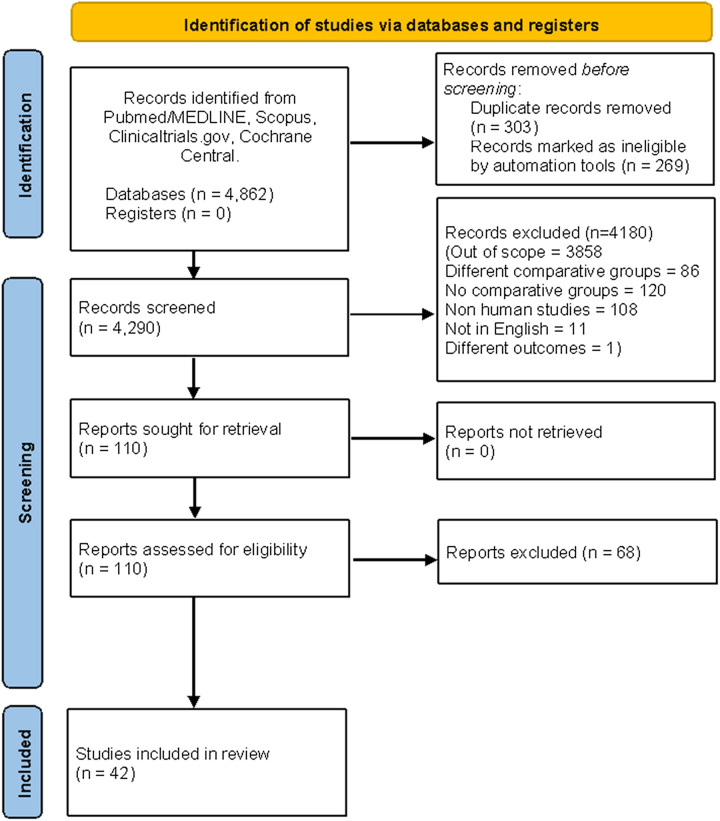
PRISMA flowchart.

A comprehensive literature search initially identified a total of 4862 articles. Following removal of duplicates and screening of titles and abstracts, full-text screening was conducted, resulting in the inclusion of 42 randomized controlled trials (RCTs) in the final analysis^19–60^. These trials involved a total of 3696 participants, with 1824 in the PRP group, 1269 in the HA group, 437 in the placebo group, and 166 in the CS group. PRP was compared with HA in 28 studies, saline in 10 studies, CS in 6 studies, and ozone in three studies. A detailed literature search is illustrated in the PRISMA flowchart (Fig. 1). SDC 3, Table 1, Supplemental Digital Content 3, http://links.lww.com/MS9/A334 provides a summary of the baseline characteristics of all studies that were included in the analysis.

**Table 1:** WOMAC pain, stiffness, function and total scores at 1-month, 3-month, 6-month and 12-month follow-up stratified by subgroups PRP versus hyaluronic acid, steroids or placebo

Outcomes	Duration in months	Studies Included (*n*)	MD	Lower 95% CI	Upper 95% CI	*P*	I^2^ (%)
PRP versus HA							
WOMAC pain	1	6	0.01	−0.13	0.15	0.86	16
	3	7	−0.35	−0.77	0.07	0.11	81
	6	10	−0.96	−1.56	−0.37	0.001	94
	12	6	−1.85	−2.46	−1.23	<0.00001	91
Overall result	—	—	−0.74	−1.02	−0.46	<0.00001	94
WOMAC stiffness	1	5	0.04	−0.30	0.37	0.84	73
	3	6	−0.14	−0.37	0.10	0.26	45
	6	8	−0.26	−0.57	0.04	0.09	81
	12	5	−0.99	−1.39	−0.60	<0.00001	79
Overall result	—	—	−0.32	−0.47	−0.17	<0.0001	88
WOMAC function	1	4	−1.38	−2.60	−0.15	0.03	80
	3	5	−1.69	−3.76	0.38	0.11	91
	6	8	−2.68	−5.04	−0.31	0.03	98
	12	5	−8.13	10.08	−6.18	<0.00001	84
Overall result	—	—	−3.52	−4.96	−2.09	<0.00001	98
WOMAC total	1	6	−1.34	−2.38	0.16	0.08	65
	3	8	−4.04	−7.58	−0.49	0.03	97
	6	12	−6.46	−9.81	−3.11	0.0002	98
	12	8	−10.44	−12.87	−8.00	<0.00001	89
Overall result	—	—	−5.80	−7.46	−4.13	<0.00001	97
PRP versus placebo							
WOMAC pain	1	6	−0.55	−1.47	0.37	0.24	64
	3	4	−3.53	−4.93	−2.12	<0.00001	80
	6	6	−3.10	−4.85	−1.36	0.005	91
Overall result	—	—	−2.24	−3.34	−1.15	<0.0001	93
WOMAC stiffness	1	7	−0.18	−0.54	0.18	0.33	54
	3	4	−1.31	−1.56	−1.07	<0.00001	0
	6	6	−1.20	−2.09	−0.31	0.008	94
Overall result	—	—	−0.83	−1.26	−0.40	0.0002	90
WOMAC function	1	7	−0.16	−1.23	0.91	0.77	6
	3	4	−7.86	−12.23	−3.50	0.0004	90
	6	6	−9.82	−19.36	−0.28	0.04	97
Overall result	—	—	−5.99	−9.40	−2.57	0.0006	95
WOMAC total	1	8	−3.24	−7.33	0.84	0.12	80
	3	6	−10.84	−18.24	−3.44	0.004	93
	6	7	−8.89	−18.79	1.01	0.08	96
	12	2	−2.89	−40.98	35.20	0.88	98
Overall result	—	—	−7.01	−11.26	−2.76	0.001	95
PRP versus CS							
WOMAC pain	6	2	−4.67	−5.47	−3.86	<0.00001	0
WOMAC total	6	2	−7.21	−9.04	−5.37	<0.00001	97

CS, corticosteroid; HA, hyaluronic acid; MD, mean difference; PRP, platelet-rich plasma; WOMAC, Western Ontario and McMaster Universities Arthritis Index.

### Quality assessment and publication bias

The quality of the studies was assessed using the Cochrane risk of bias tool for RCTs, and the results indicated low risk of bias in majority of studies, as shown in (SDC 3, Table 2, Supplemental Digital Content 3, http://links.lww.com/MS9/A334). Egger’s test revealed a significant publication bias in almost all the outcomes as demonstrated in SDC 3, Table 3, Supplemental Digital Content 3, http://links.lww.com/MS9/A334. Funnel plots for publication bias have been shown in SDC 4, Figures S1-3, Supplemental Digital Content 4, http://links.lww.com/MS9/A335.

**Table 2:** VAS pain, IKDC and KOOS scores at one, three, six- and 12-months follow-up stratified by subgroups PRP versus HA, CS or placebo

Outcomes	Duration in months	Studies Included (*n*)	MD	Lower 95% CI	Upper 95% CI	*P*	I^2^ (%)
PRP versus HA							
VAS pain	1	5	−0.19	−0.33	−0.05	0.008	0
	3	6	−0.55	−1.30	0.21	0.16	83
	6	6	−0.73	−1.89	0.43	0.22	96
	12	5	−1.06	−2.39	0.28	0.12	97
Overall results	—	—	−0.65	−1.24	−0.06	0.03	97
IKDC							
	1	3	2.15	−1.04	5.74	0.19	0
	2	4	0.46	−2.31	3.23	0.75	0
	3	3	4.96	1.70	8.22	0.003	0
	6	7	4.59	1.96	7.23	0.0006	40
	12	4	3.93	−0.83	8.69	0.11	42
Overall results	—	—	3.31	1.89	4.73	<0.00001	23
PRP versus CS							
VAS pain	1	3	−0.45	−0.93	0.04	0.07	0
	3	3	−1.40	−3.01	−0.21	0.09	76
	6	3	−1.78	−2.74	−0.82	0.0003	42
	12	2	−1.19	−3.06	−0.69	0.22	88
Overall results	—	—	−1.11	−1.64	−0.59	<0.0001	68
KOOS pain							
	1	2	−0.28	−7.22	6.66	0.94	32
	3	2	5.79	0.40	11.18	0.04	19
	6	2	11.32	−1.66	24.30	0.09	79
Overall results	—	—	4.99	−1.65	11.64	0.14	83
PRP versus placebo							
VAS pain	1	3	−1.17	−1.57	−0.77	<0.00001	0
	3	3	−2.70	−3.08	−2.32	<0.00001	0
	6	3	−1.41	−3.92	1.10	0.27	98
Overall results	—	—	−1.74	−2.68	−0.80	0.0003	95

CS, corticosteroid; HA, hyaluronic acid; IKDC, International Knee Documentation Committee; KOOS, Knee Injury and Osteoarthritis Outcome Score; MD, mean difference; PRP, platelet-rich plasma; VAS, Visual Analog Scale.

### PRP versus HA

#### WOMAC total

**Figure 2:**
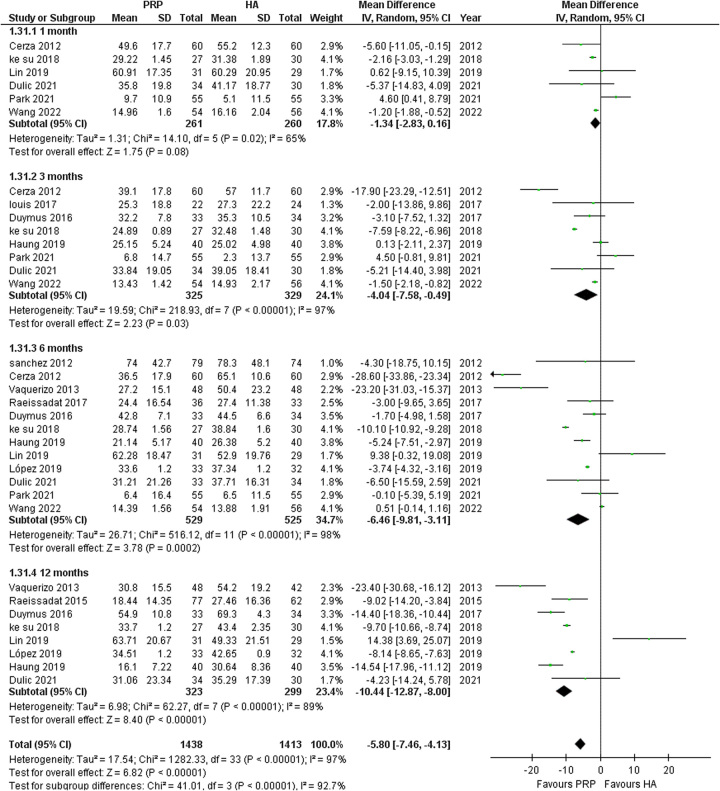
Forest plots for the subgroup analysis of platelet-rich plasma (PRP) versus hyaluronic acid (HA) for the outcomes of Western Ontario and McMaster Universities Arthritis Index total at 1, 3, 6, and 12 months.

Thirteen studies were analyzed to evaluate the difference between PRP and HA in terms of the WOMAC total scores. The findings of the analysis demonstrated a notable and statistically significant enhancement associated with PRP treatment (MD: −5.80; 95% CI: −7.46 to −4.13; *P*<0.00001). Nevertheless, due to the substantial heterogeneity observed (*I* ^2^=97%), we performed a subgroup analysis focusing on studies that conducted follow-ups at 1, 3, 6, and 12 months, assessing the WOMAC total score. Our subgroup analysis revealed a considerable significant reduction in WOMAC total at 12 months follow-up (MD: −10.44; 95% CI: −12.87 to −8.00; *I* ^2^=89% *P≤*0.00001) as compared to 3-month and 6-month follow-up (MD: −4.04; 95% CI: −7.58 to −0.49; *I* ^2^=97%, *P*=0.03 and MD: −6.46; 95% CI: −9.81 to −3.11; *I* ^2^=98%, *P*=0.0002 respectively), with no statistically significant difference at 1 month. (Fig. 2) To further investigate the results for the outcome of WOMAC total at 1 month, a sensitivity analysis was conducted by excluding the study Park *et al.* ^25^. This analysis revealed a statistically significant reduction in WOMAC total with PRP (MD: −1.75; 95% CI: −2.64 to −0.87; *I*²=31%; *P*=0.0001). (SDC 4, Figure S4, Supplemental Digital Content 4, http://links.lww.com/MS9/A335).

#### WOMAC pain

Similarly, the WOMAC pain subscale was analyzed using data from 12 studies. Analysis of these studies demonstrated a statistically significant reduction in WOMAC pain (MD: −0.74; 95% CI: −1.02 to −0.46; *I* ^2^=94%; *P≤*0.00001). Further subgroup analysis for WOMAC pain at 1, 3, 6 and 12 months revealed a statistically significant and nearly equivalent reduction in WOMAC pain scores at 6 and 12 months (MD: −0.96; 95% CI: −1.56 to −0.37; *I* ^2^=94%; *P*=0.001 and MD: −0.74; 95% CI: −1.02 to −0.46; *I* ^2^=94%; *P≤*0.00001 respectively) while no significant difference was observed at 1 and 3 months. (Table 1; SDC 4, Figure S5, Supplemental Digital Content 4, http://links.lww.com/MS9/A335) Leave-one-out analysis was performed for WOMAC pain at 3 months, exclusion of study Cole *et al.* ^45^. resulted in reduction of heterogeneity from 84% to 6% and a statistically significant difference between groups (MD: −0.30; 95% CI: −0.46 to −0.13; *I*²=6%; *P*=0.0004). (SDC 4, Figure S6, Supplemental Digital Content 4, http://links.lww.com/MS9/A335).

#### WOMAC stiffness

Analysis of WOMAC stiffness subscale also revealed a statistically significant reduction in stiffness scores (MD: −0.32; 95% CI: −0.47 to −0.17; *I* ^2^=88%; *P≤*0.0001). Further subgroup analysis of different time intervals revealed a significant reduction in stiffness score at 12 months only (MD: −0.99; 95% CI: −1.39 to −0.60; I^2^=79%; *P≤*0.00001). At 1-month, 3-month and 6-month follow-up, there was a non-significant reduction in stiffness scores. (Table 1; SDC 4, Figure S7, Supplemental Digital Content 4, http://links.lww.com/MS9/A335)

#### WOMAC function

WOMAC subscale of function resulted in a significant improvement, favoring PRP over HA (MD: -3.52; 95% CI: −496 to −2.09; *I* ^2^=98%; *P≤*0.00001). Further subgroup analysis revealed greatest improvement in function at 12 months (MD: −8.13; 95% CI: −10.08 to −6.18; *I* ^2^=84%; *P≤*0.00001) as compared to 1-month and 6-month (MD: −1.38; 95% CI: −2.60 to −0.15; *I* ^2^=80%; *P*=0.03 and MD: −2.68; 95% CI: −5.04 to −0.31; *I* ^2^=98%; *P*=0.03). (Table 1; SDC 4, Figure S8, Supplemental Digital Content 4, http://links.lww.com/MS9/A335).

#### VAS pain

**Figure 3:**
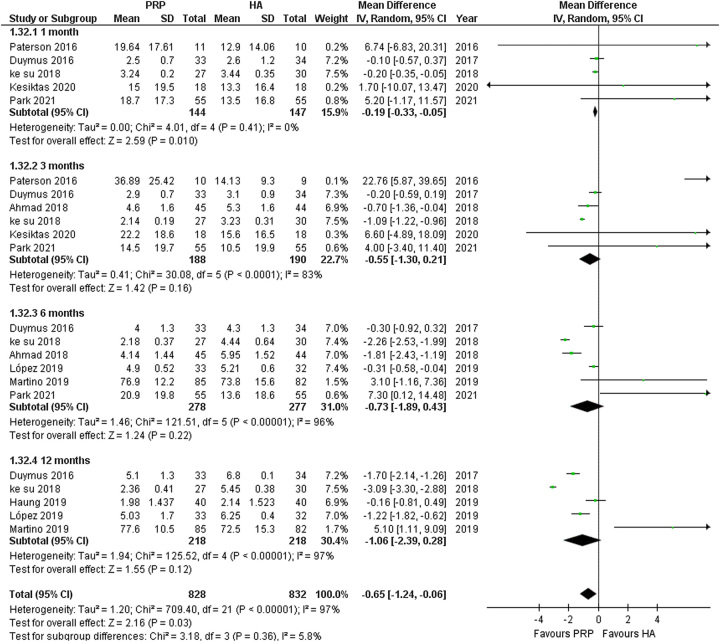
Forest plots for the subgroup analysis of platelet-rich plasma (PRP) vs hyaluronic acid (HA) for the outcomes of Visual Analog Scale pain at 1, 3, 6, and 12 months.

The analysis of VAS pain revealed a statistically significant reduction in pain score with PRP (MD: −0.65; 95% CI: −1.24 to −0.06; *I* ^2^=97%; *P*=0.03). Further subgroup analysis of VAS pain at 1-month, 3-month, 6-month, and 12-month follow-ups demonstrated a significant reduction in VAS pain score at only 1-month follow-up favoring PRP against HA (MD: −0.19; 95% CI: −0.33 to −0.05; *I* ^2^=0%; *P*=0.010), with rest being non-significant. (Fig. 3) Additionally, sensitivity analysis for the outcome of VAS pain at 3 months after excluding Paterson *et al.* ^54^. and Duymus *et al.* ^46^. revealed a significant result with reduced heterogeneity (MD: −0.92; 95% CI: −1.45 to −0.39; *I*²=37%; *P*=0.0007). (SDC 4, Figure S9, Supplemental Digital Content 4, http://links.lww.com/MS9/A335).

#### IKDC

An additional outcome IKDC was reported centred on impact of PRP versus HA that revealed a significant improvement in IKDC scores with PRP (MD: 3.31; 95% CI 1.89 to 4.73; I^2^=23%; *P≤*0.00001). Subgroup analysis at different intervals of 1, 2, 3, 6 and 12th month follow-up revealed no significant difference in 1st, 2nd and 12th month. However, at 3rd and 6th month PRP proved to be statistically significant over HA (MD: 4.96; 95% CI: 1.70 to 8.22; I^2^=0%; *P*=0.003 and MD: 4.59; 95% CI 1.96 to 7.23; I^2^=40%; *P*=0.0006). Low to moderate heterogeneity was observed. (Table 2; SDC 4, Figure S10, Supplemental Digital Content 4, http://links.lww.com/MS9/A335).

### PRP versus CS

#### WOMAC total

Two studies recorded WOMAC total scores that demonstrated a significant improvement with PRP as compared to CS (MD: −8.83; 95% CI −16.77 to −0.89; *P*=0.03; I^2^=97%). Subgroup analysis of two studies reporting WOMAC total at 6 months did not reveal a significant difference between the two treatment groups. At 3- and 12-months follow-up subgroup analysis could not be performed due to a limited number of studies. (Table 1; SDC 4, Figure S11, Supplemental Digital Content 4, http://links.lww.com/MS9/A335).

#### WOMAC pain

Two studies recorded WOMAC pain scores, that revealed a significant improvement with PRP as compared to CS (MD: -8.06; 95% CI: −13.62 to −2.51: *P*=0.004; I2=96%). Further subgroup analysis revealed a statistically significant reduction in pain scores at 6 months with PRP use (MD: −4.67; 95% CI; −5.47 to −3.86; *P≤*0.00001; I^2^=0%). (Table 1; SDC 4, Figure S12, Supplemental Digital Content 4, http://links.lww.com/MS9/A335).

#### VAS pain

Four studies analyzed the VAS pain score and found a significant improvement with PRP (MD: −1.11; 95% CI: −1.64 to −0.59; *P≤*0.0001; I^2^=68%). Subgroup analysis at intervals of 1-, 3-, 6- and 12-months revealed a significant improvement in pain score at 6 months only (MD: −1.78; 95% CI: −2.74 to −0.82; *P* = 0.0003; I^2^=42%) and non-significant reduction in pain scores at 1, 3, and 12 months. (Table 2; SDC 4, Figure S13, Supplemental Digital Content 4, http://links.lww.com/MS9/A335).

#### KOOS pain

Additionally, KOOS pain was addressed by 2 trials that did not reveal any significant difference between the treatment groups. However, subgroup analysis at different time intervals revealed a significant improvement with steroids at 3 months (MD: 5.79; 95% CI 0.40 to 11.18; *P*=0.04; I^2^=19%) whereas, follow-ups at 1st and 6th month were statistically insignificant. (Table 2; SDC 4, Figure S14, Supplemental Digital Content 4, http://links.lww.com/MS9/A335).

### PRP versus Placebo

#### WOMAC total

Eight studies evaluated the outcome of WOMAC total in OA patients and found a significant reduction in WOMAC total score with PRP (MD: −7.01: 95% CI: −11.26 to −2.76; I^2^=95%: *P*=0.001). Considering significant heterogeneity, subgroup analysis for different follow-up periods was performed that found a significant reduction in WOMAC total scores at 3 months (MD: −10.84; 95% CI: −18.24 to −3.44; I^2^=93%; *P*=0.004) and a non-significant reduction at 1 and 6 months. (Table 1; SDC 4, Figure S15, Supplemental Digital Content 4, http://links.lww.com/MS9/A335). Further, sensitivity analysis for the outcome of WOMAC total at 6 months after excluding Duymus *et al.* ^46^. and Lin *et al.* ^48^. revealed a significant difference between both treatment groups (MD: −7.60; 95% CI: −10.75 to −4.44; *P≤*0.00001) and a reduction in heterogeneity from 96% to 43%. (SDC 4, Figure S16, Supplemental Digital Content 4, http://links.lww.com/MS9/A335).

#### WOMAC pain

Six studies addressed WOMAC pain subscale, analysis of these studies demonstrated a significant improvement with PRP (MD: −2.24: 95% CI: −3.34 to −1.15; I^2^=93%; *P≤*0.0001). Subsequent subgroup analysis demonstrated an insignificant difference at 1-month follow-up. On the other hand, third and sixth month follow-up periods documented a statistically significant and almost equivalent reduction in WOMAC pain (MD: -3.53: 95% CI: −4.93 to −2.12; I^2^=80%; *P≤*0.00001 and MD: −3.10; 95% CI: −4.85 to −1.36; I^2^=91%; *P*=0.0005 respectively). (Table 1; SDC 4, Figure S17, Supplemental Digital Content 4, http://links.lww.com/MS9/A335) Sensitivity analysis after removing Dorio *et al.* ^27^. for the outcome of WOMAC pain at 3 months and both Duymus *et al.* ^46^. and Dorio *et al.* ^27^. for the outcome of WOMAC pain at 6 months demonstrated reduced heterogeneity and a statistically significant difference (MD: −4.31; 95% CI: −5.14 to −3.48; I²=38%; *P*≤0.00001 and MD: −2.93; 95% CI: −3.85 to −2.01; I²=50%; *P≤*0.00001 respectively). (SDC 4, Figure S18, Supplemental Digital Content 4, http://links.lww.com/MS9/A335)

#### WOMAC stiffness

Analysis of 7 studies that reported WOMAC stiffness depicted significant reduction in stiffness score with PRP (MD: −0.83; 95% CI: −1.26 to −0.40; I^2^=90%; *P*=0.0002). In regards to WOMAC stiffness subgroup analysis, at third and sixth month follow-up PRP was favourable with significant stiffness reduction (MD: −1.31; 95% CI: −1.56 to −1.07; I^2^=0%; *P≤*0.00001 and MD: −1.20; 95% CI: −2.09 to −0.31; I^2^=94%; *P*=0.008 respectively), meanwhile stiffness subscore remained non-significant at 1 month. (Table 1; SDC 4, Figure S19, Supplemental Digital Content 4, http://links.lww.com/MS9/A335).

#### WOMAC function

Analysis of WOMAC function showed significant improvement, results inclining towards PRP over Placebo (MD: −5.99; 95% CI: −9.40 to −2.57; I^2^=95%; *P*=0.0006). Additionally, on subgroup analysis of WOMAC function at 1-month follow-up, there were no noticeable improvements between the treatments. A statistically significant change was observed in 3rd and 6th months follow-up in terms of WOMAC function with greater improvement at 6 months (MD: −7.86; 95% CI: −12.23 to −3.50; I^2^=90%; *P*=0.0004 and MD: −9.82; 95% CI: −19.36 to −0.28; I^2^=97%; *P*=0.04 respectively) (Table 1; SDC 4, Figure S20, Supplemental Digital Content 4, http://links.lww.com/MS9/A335).

#### VAS pain

Following analysis for VAS pain a statistically significant subgroup effect was observed (MD: −1.74; 95% CI: −2.68 to −0.80; I^2^=95%; *P*=0.0003). Moreover, on subgroup analysis at different intervals no significant difference was noted at 6 months follow-up, meanwhile the subgroup analysis at one- and 3-months follow-ups showed statistically significant difference (MD: −1.17; 95% CI: −1.57 to −0.77; I^2^=0%; *P≤*0.00001 and MD: −2.70; 95% CI: −3.08 to −2.32; I^2^=0%; *P≤*0.00001). (Table 2; SDC 4, Figure S21, Supplemental Digital Content 4, http://links.lww.com/MS9/A335).

## Discussion

Current Meta-analysis including 42 trials involving 3696 patients suggests that PRP is an effective treatment for knee osteoarthritis when compared with HA, CS and placebo. The recent evidence based clinical practice guidelines from the AAOS on appropriate use criteria for the management of knee osteoarthritis provided treatment recommendations for specific patient scenarios. In the majority of cases, PRP was rated as “Rarely Appropriate,” while intra-articular CS was considered “Appropriate.“^14^ In an effort to provide symptomatic relief and postpone surgery, intra-articular CS injections are frequently prescribed prior to secondary care referral. While these injections have shown temporary improvement in pain scores among osteoarthritic patients, they are also associated with side effects^61^. Thus, our findings have the potential to provide valuable decision support in favour of PRP for the development of future guidelines.

Inflammation plays a significant role in the development and progression of osteoarthritis, contributing to joint symptoms and disease advancement^62^. Anti-inflammatory approaches can effectively counteract this key mechanism of disease progression. Blood derivatives such as PRP have the potential to exert broad influences on the joint environment. PRP can affect synoviocytes, meniscal cells, and mesenchymal stem cells, thereby modulating various cellular activities^63–65^. Additionally, the chemo-attractant properties of PRP can attract other beneficial cells to participate in the overall therapeutic effect^63^. This multifaceted action of PRP may result in anabolic effects, down-regulation of joint inflammation, and positive modulation of chondrocyte apoptosis^66^. Consequently, PRP can offer clinical benefits by improving symptoms and function and potentially slowing down the degenerative processes, even though it may not directly regenerate hyaline cartilage^67^.

Our findings align with previous meta-analyses, indicating that PRP outperforms HA, CS, and placebo in terms of efficacy^61,67,68^. Specifically, when compared to CS injections, PRP demonstrates greater efficacy in reducing WOMAC pain and VAS pain outcomes, with the most significant improvement observed at 6 months. A Cochrane review examining the use of CS injections for knee OA supports our results, stating that the effectiveness of the injection diminishes over time, with no sustained effect at 6-month post-injection^61,69^. Additionally, subgroup analysis showed significant improvement with steroids at 3 months, likely due to their quick and symptomatic relief. However, the limited number of studies included in this analysis necessitates further research to validate these findings. Additionally, WOMAC pain, stiffness, and function in the PRP group showed greatest improvement in the 12th month follow-up. This is supported by previous evidence by Shen *et al.* ^70^. and Filardo *et al.* ^67^. who suggested a sustained effect following PRP injections of up to 12 and even 24 months^61^.

The research findings demonstrate that PRP showed greater improvement in relieving pain in the knee joint compared to CS, as indicated by the significant differences in WOMAC parameters (total, pain) and VAS pain outcomes. Both PRP and corticosteroids have anti-inflammatory properties. However, PRP exerts a more targeted and controlled anti-inflammatory response. It reduces inflammation by modulating the immune response and increasing angiogenesis and re-epithelialization^71^. Whereas corticosteroids broadly suppress the immune system providing only temporary pain relief^72^. Additionally, PRP has the potential to modify the underlying disease process in knee joint conditions, such as osteoarthritis^14^. By promoting tissue repair and regeneration, PRP may slow down the progression of the disease and prevent further joint damage. Corticosteroids do not have disease-modifying properties and primarily address symptom management

Significant improvement in pain relief was observed with PRP treatment compared to intra-articular injections of HA for several outcomes such as WOMAC parameters (total, pain, stiffness, function), VAS pain and IKDC. PRP contains a high concentration of growth factors (GF), cytokines, and other bioactive molecules that have regenerative effects on damaged tissues. These substances stimulate tissue repair, reduce inflammation, and promote healing in the knee joint^73^. This regenerative capacity of PRP may lead to more effective pain relief compared to HA. Additionally, subgroup analysis revealed the greatest improvement in function at the 12-month follow for WOMAC scores (total, stiffness and function). The beneficial effects of PRP treatment may persist over a longer duration compared to HA. PRP stimulates tissue healing and regeneration, leading to sustained pain relief and functional improvements. HA, being primarily a lubricant, may provide temporary relief but may not have the same long-term impact as PRP^74^.

Different PRP formulations exist, varying in concentrations of blood cells, plasma, and GFs. The role of leucocytes in PRP remains debated, with conflicting findings regarding their pro-inflammatory effects^67^. The only available comparative trial showed similar outcomes between leucocyte-rich (LR) and leucocyte-poor (LP) PRP formulations^75^. According to recent meta-analysis results, it was found that three injections of PRP had a significantly greater effect compared to a single injection, and LR-PRP demonstrated higher efficacy than LP-PRP^67^. However, due to the limited available information, additional research is required to further substantiate these findings^14^. It is worth noting that the reporting of PRP composition is often inadequate, and inconsistent definitions further complicate the analysis. Thus, additional high-level studies that compare specific PRP formulations are necessary to draw reliable conclusions^67^. In the management of knee osteoarthritis, the concentration of platelets, the volume of PRP injected, and the treatment protocol can be customized based on the severity and characteristics of the knee osteoarthritis. This individualized approach may contribute to better outcomes compared to the standardized intra-articular treatment.

### Limitations

One potential limitation is the presence of high unexplained heterogeneity. This could be attributed to factors such as the method of preparation, centrifugation process, concentration of leucocytes, and dosage of PRP. These variations have the potential to generate distinct biological effects of PRP and HA, leading to varying physiological responses in patients. An author’s review provides guidance and recommendations on the key components that should be included in a standardized PRP protocol^76^. There is also substantial heterogeneity among the patients included in the meta-analyses regarding patient age, sex, BMI. Additionally, our selection of studies was limited to those published in the English language, which introduces the possibility of a bias related to language or culture. Lastly, the significant results of Egger’s test indicate that publication bias may have influenced our meta-analysis findings. This suggests that studies with positive or statistically significant results are more likely to be published, which could result in an overestimation of the treatment effect. Caution is advised when interpreting our results, and additional studies are required to gain a more thorough and unbiased understanding of the topic.

## Conclusions

In conclusion, PRP offers symptomatic relief, potentially slows down disease progression, and has sustained effects up to 12 months. It provides better pain relief and functional improvement than CS and HA injections. PRP’s effectiveness is contributed to by its anti-inflammatory and regenerative properties. However, additional research is required to investigate the function of leucocytes in PRP formulations. Individualizing PRP treatment based on disease severity has the potential to improve outcomes. In general, PRP has the potential to influence future knee osteoarthritis treatment guidelines and decision-making.

## Ethical approval

Not applicable.

## Consent

Not applicable.

## Sources of funding

Not applicable.

## Author contributions

S.K. and A.A. contributed to the study conception and design. Deepak, M.S.Z., and M.Q. performed literature search and data extraction. Deepak and L.U.M. analysed the data. A.A., Deepak, Z.F. and R.A.N. drafted the manuscript. S.K. and P.B. critically revised the manuscript for important intellectual content. All authors have read and approved the final manuscript and take full responsibility for the accuracy and integrity of all aspects of the work.

## Conflicts of interest disclosure

The authors declare no conflict of interest.

## Research registration unique identifying number (UIN)

Not applicable.

## Guarantor

Saad Khalid.

## Data availability

Available upon request from the corresponding author.

## Provenance and peer review

Not commissioned, externally peer-reviewed.

## Supplementary Material

SUPPLEMENTARY MATERIAL
