# Supplementary material for: Comparative effectiveness of intra-articular therapies in knee osteoarthritis: a meta-analysis comparing platelet-rich plasma (PRP) with other treatment modalities
Source: Ann Med Surg (Lond). 2023 Dec 15;86(1):361–72. doi: 10.1097/MS9.0000000000001615 (PMC10783230; doi:10.1097/MS9.0000000000001615)
Supplement: SUPPLEMENTARY MATERIAL [file ms9-86-361-s003.docx]

**SUPPLEMENTARY TABLES**

| Study | Type of  Control | Knee/Patients  included | | | Total Patients | Sex, n (M/F) | | Age (yr), Mean ± SD | | | BMI (kg/m2), Mean ± SD | | | Total WOMAC baseline, Mean ± SD | | | VAS pain score baseline, Mean ± SD | |
| --- | --- | --- | --- | --- | --- | --- | --- | --- | --- | --- | --- | --- | --- | --- | --- | --- | --- | --- |
|  |  | **PRP** | **Control** | |  | **PRP** | **Control** | **PRP** | | **Control** | **PRP** | | **Control** | **PRP** | | **Control** | **PRP** | **Control** |
| Wang 2022 [1] | HA | 54 | | 56 | 110 | 12/42 | 16/40 | 61.87 ± 5.46 | 63.00 ± 5.33 | | 24.07 ± 3.35 | 24.02 ± 2.39 | | 18.74 ± 1.85 | 18.23 ± 1.80 | | NR | NR |
| Kesiktas 2022 [2] | Peptide/  HA | 18 | | Peptide: 18  HA: 18 | 54 | 2/16 | Peptide: 4/14  HA: 4/14 | 52. 7 ± 8.3 | Peptide: 59.7 ± 6.8  HA: 55 1 ± 10.3 | | 28.3 ± 4.4 | Peptide: 31.5 ± 4.6  HA:31 .0 ± 4.9 | | 28.3 ± 4.4 | Peptide: 28.3 ± 4.4  HA: 28.3 ± 4.4 | | 41.1 ± 22.2 | Peptide: 25.0 ± 25.7  HA: 25.0 ± 25.7 |
| Huang 2022 [3] | PRP+HA | PRP+ Artz: 48 | | PRP+  HYAJOINT Plus: 47 | 95 | PRP+ Artz: 17/31 | PRP+  HYA  JOINT Plus:  17/30 | PRP  + Artz: 61.9±8.8 | PRP+  HYA  JOINT Plus: 61.0±8.1 | | PRP  + Artz: 24.5  ±3.8 | PRP+  HYA  JOINT Plus:  25.4±4.4 | | PRP + Artz: 5.9±3.8 | PRP + HYAJOINT Plus:  6.0±3.2 | | PRP + Artz: 42.3 ± 11.2 | PRP + HYAJOINT Plus: 39.7 ± 10.3 |
| Finogejevs 2022 [4] | CS | 20 | | 20 | 40 | 17/3 | 15/5 | 66.5 ± 8.6 | 70.1 ± 9.1 | | 28.6 ± 5.0 | 30.5 ± 5.8 | | NR | NR | | 6.1 ± 1.2 | 6.0 ± 1.4 |
| Bennell 2021[5] | Saline | 144 | | 144 | 288 | 59/85 | 60/84 | 62.2 ± 6.3 | 61.6 ± 6.6 | | 29.0 ± 3.7 | 29.6 ± 4.5 | | NR | NR | | NR | NR |
| Raeissadat 2021 [6] | HA/Ozone | 52 | | HA:51  Ozone:48 | 200 | 13/39 | HA:  14/37  Ozone: 12/36 | 56.09 ± 6.0 | HA: 57.91 ± 6.7  Ozone:  57.60 ± 6.1 | | 27.41 ± 2.6 | HA: 27.46± 2.2  Ozone: 27.01± 1.9 | | 42.73 ± 7.7 | HA:  42.75 ± 11.1  Ozone:  42.79 ± 8.2 | | 7.92 ± 1.0 | HA:  8.22 ± 1.1  Ozone:  8.10 ± 1.0 |
| Park 2021 [7] | HA | 55 | | 55 | 110 | 16/39 | 8/47 | 60.6 ± 8.2 | 62.3 ± 9.6 | | 25.5 ± 2.2 | 25.9 ± 2.8 | | NR | NR | | 59.0± 9.9 | 55.2 ± 9.5 |
| Dulic 2021 [8] | HA | 34 | | 30 | 175 | 15/19 | 13/17 | 58.8± 11.2 | 59.4 ± 14.0 | | 28.61 ± 4.53 | 29.98 ± 5.24 | | NR | NR | | 6.94 ± 1.79 | 6.90 ± 1.81 |
| Dorio 2021 [9] | saline | PRP: 20  Plasma: 21 | | 21 | 62 | PRP: 1/19  Plasma: 2/19 | 2/19 | PRP:66.4 ± 5.6  Plasma: 66.1 ± 7.5 | 62.5 ± 8.1 | | PRP: 28.3 ± 4.1  Plasma: 28 ± 3.1 | 27.6 ± 3.8 | | PRP: 52.9 ± 15.5 Plasma: 46.71± 4.3 | 52.3 ±15.9 | | PRP: 6.1 ± 1.6  Plasma: 5.9 ± 1.4 | 6.6 ± 1.4 |
| Tucker 2021 [10] | saline | 10 | | 6 | 16 | 8/2 | 2/4 | 57.5±1.8 | 57.2±3.9 | | 30.9±1.5 | 29.1±2.1 | | NR | NR | | 48.7 ± 9.8 | 37.9 ± 9.2 |
| Sdeek 2021 [11] | HA | 95 | | 94 | 189 | 15/80 | 16/78 | 60.2 | 59.5 | | 27.9 | 27.1 | | 66.5 | 66.9 | | 40.9 | 60.3 |
| Elik 2020 [12] | saline | 30 | | 27 | 57 | 1/29 | 3/24 | 61.30 ± 7.91 | 60.19 ± 6.80 | | 30.37 ± 4.47 | 30.70 ± 3.97 | | 11.13 ± 4.27 | 12.00 ± 2.91 | | 3.87 ± 2.14 | 4.93 ± 1.68 |
| Freire 2020 [13] | CS | 25 | | 25 | 50 | 4/21 | 4/21 | 64.15 ± 8.02 | 60.21 ± 5.92 | | NR | NR | | 73.94 | 63.93 | | NR | NR |
| Martino 2019 [14] | HA | 85 | | 82 | 167 | 53/32 | 47/35 | 52.7± 13.2 | 57.5 ± 11.7 | | 27.2± 7.6 | 26.8± 4.3 | | NR | NR | | 72.7 ± 12.3 | 71.2 ± 13.3 |
| Gaballa 2019 [15] | Ozone | 20 | | 20 | 60 | 5/15 | 4/16 | 53.6 ± 4.6 | 56.3 ±  4.4 | | NR | NR | | 49 ± 7.5 | 52 ± 6.8 | | 6.8 ± 1.1 | 6.9 ± 1.2 |
| Ghai 2019 [16] | saline | 10 | | 10 | 20 | 25%/  75% | 25%/  75% | 49.8 ± 9.42 | 49.8 ± 9.42 | | 67 ± 9.56 | 67 ± 9.56 | | 37.5 ± 3 | 26.65 ± 2.9 | | 8.40 ± 0.883 | 7.15 ± 0.93 |
| Huang 2019 [17] | HA & steroid | 40 | | HA: 40  Steroid: 40 | 120 | 25/15 | HA: 19/21  Steroid: 21/19 | 54.5 ± 1.2 | HA: 54.8 ± 1.1  Steroid: 54.3 ± 1.4 | | 25.23 ± 4.15 | HA: 24.51 ± 3.09  Steroid:  24.56 ± 3.62 | | 48.19 ± 4.96 | HA: 47.23 ± 5.37  Steroid: 46.58 ± 5.74 | | 4.57 ± 0.610 | HA: 4.54 ± 0.596  Steroid: 4.64 ± 0.543 |
| Rahimzadeh 2018 [18] | PRL | 21 | | 21 | 42 | 10/11 | 11/10 | 65.5± 6.64 | 64.3 ± 5.31 | | 28.6± 1.8 | 28.3 ± 1.9 | | 67.9 ± 7.3 | 67.1 ± 7.9 | | NR | NR |
| Sanchez [19] | HA | 79 | | 74 | 153 | 33/46 | 29/45 | 60.5 ±  7.9 | 58.9 ± 8.2 | | 27.9 ± 2.9 | 28.2 ± 2.7 | | 121.8 ± 44.4 | 115.6 ± 45.1 | | NR | NR |
| Guvendi 2018 [20] | CS | Single PRP: 19 Three PRP: 14 | | 17 | 50 | Single PRP: 1/18 Three PRP: 1/13 | 2/15 | Single PRP: 62.3±1.6 Three PRP: 60.4±1.7 | 62.8 ± 1.7 | | Single PRP: 31.4±  0.7 Three PRP: 31.0±  1.0 | 31.1±1.0, | | Single PRP: 58.1 ± 3.3 Three PRP: 62.9 ± 4.2 | 59.7 ± 3.2 | | NR | NR |
| Nabi 2018 [21] | CS | 33 | | 34 | 67 | 5/28 | 7/27 | 59.09 ± 7.79 | 58.55 ± 8.79 | | 28.4 ± 2.78 | 27.78 ± 3.29 | | NR | NR | | 7.36 ± 0.92 | 7.12 ± 1.29 |
| Louis 2018 [22] | HA | 24 | | 24 | 48 | 14/10 | 11/13 | 53.2 ± 11.7 | 48.5 ±11.5 | | 25.6 ± 2.9 | 27.0 ± 2.9 | | NR | NR | | 4.8 ± 2.3 | 5.0 ± 2.4 |
| Ahmad 2018 [23] | HA | 45 | | 44 | 89 | 14/31 | 15/30 | 56.2 ± 6.8 | 56.8 ± 7.4 | | 26.7 ± 3.6 | 26.5 ± 3.5 | | NR | NR | | 5.8 ± 1.9 | 6.1 ± 1.7 |
| Lopez 2018 [24] | HA/  NSAID | 33 | | HA: 32  NSAID: 33 | 98 | 16/17 | HA: 15/17  NSAID: 16/17 | 56.15±  3.001 | HA: 56.63± 2.9  NSAID: 57.42±3.1 | | 24.9±  0.32 | HA: 24.9±  0.41  NSAID: 25.2± 0.48 | | 42.57 ± 7.3 | HA: 42.62 ± 7.3 NSAID: 42.66 ± 7.8 | | 6.15 ± 1.1 | HA: 6.06 ± 0.9  NSAID: 6.15 ± 1.2 |
| Su 2018 [25] | HA | 27 | | 30 | 82 | 10/17 | 12/18 | 50.67 ± 8.70 | 53.13 ± 6.41 | | 28.19 ± 1.31 | 28.69 ± 1.13 | | 50.15 ± 1.10 | 49.88± 1.54 | | 7.09 ± 0.31 | 7.04 ± 0.33 |
| Duymus 2017 [26] | HA/ Ozone | 33 | | HA:34  Ozone:35 | 102 | 1/32 | HA: 1/33  Ozone: 4/31 | 60.4 ± 5.1 | HA: 60.3 ± 9.1  Ozone: 59.4 ± 5.7 | | 27.6 ± 4.6 | HA:  28.4 ± 3.6  Ozone: 27.6 ± 4.4 | | 76.1 ± 9.4 | HA: 77.0 ± 2.5  Ozone:76.0 ± 11.9 | | 7.4± 1.0 | HA:8.3 ±0.4  Ozone:  7.2 ± 1.1 |
| Jubert 2017 [27] | CS | 35 | | 30 | 65 | 12/23 | 6/24 | 65.56 ± 8.6 | 68 ± 7.17 | | 31.20 ± 4.36 | 30.98 ± 4.16 | | NR | NR | | 75.14 ± 10.11 | 75.00 ± 9.38 |
| Cole 2017 [28] | HA | 49 | | 50 | 99 | 28/21 | 20/30 | 55.9 ± 10.4 | 56.8 ± 10.5 | | 27.4 ± 3.9 | 29.0 ± 6.4 | | 7.00 ± 0.53 | 7.52 ± 0.58 | | 57.2 ± 00 | 62.9 ± 00 |
| Görmeli 2017 [29] | HA/saline | PRP 3 times: 39 PRP 1 time: 44 | | HA: 39  Saline: 40 | 118 | PRP 3 times: 16/23  PRP 1 times: 19/25 | HA: 17/22  Saline: 20/20 | PRP 3 times: 53.7 ± 13.1  PRP 1 times:  53.8 ± 13.4 | HA: 53.5 ± 14 saline 52.8 ± 12.8 | | PRP3 : 28.7 ± 4.8  PRP 1: 28.4 ± 4.4 | HA:29.7 ± 3.7  Saline: 29.5 ± 3.2 | | NR | NR | | NR | NR |
| Lin 2017 [30] | saline/ HA | 31 | | HA: 29  Saline: 27 | 87 | 34.48%/65.52% | HA: 34.48%/65.52%  Saline:  37.03%/62.96% | 61.17 ± 13.08 | HA:  62.53 ± 9.9  Saline:  62.23 ± 11.71 | | 23.98 ± 2.62 | HA: 26.26 ± 2.99  Saline: 24.98 ± 3.12 | | NR | NR | | NR | NR |
| Lisi 2017 [31] | HA | 30 patients  31 knees | | 28 patients  31 knees | 58 Patients  62 Knees | 20/10 | 16/12 | 53.5±  15.1 | 57.1 ± 10.0 | | NR | NR | | NR | NR | | 6.28 ± 0.59 | 5.40 ± 0.36 |
| Raeissadat 2017 [32] | HA | 36 | | 33 | 69 | 7/29 | 6/27 | 57.0 ±  7.18 | 59.5 ± 7.54 | | 28.6 ± 2.82 | 27.5±2.9 | | 42.9 ± 13.51 | 38.8 ± 12.62 | | 7.8 ± 1.78 | 7.4 ± 1.48 |
| Smith 2016 [33] | Saline | 15 | | 15 | 30 | 5/10 | 6/9 | 53.53 ± 8.22 | 46.60 ± 9.37 | | 29.53 ± 6.89 | 27.47 ± 4.78 | | 47 (41-53) * | 46 (40-53)* | | NR | NR |
| Heredia 2016 [34] | HA | 27 | | 26 | 53 | 12/15 | 9/17 | 66.3 ± 8.3 | 61.5±8.6 | | 29.0 ± 5.5 | 30.4 ± 4.9 | | NR | NR | | 15 | 15 |
| Lana 2016 [35] | HA | 36 | | 36 | 72 | 7/29 | 3/33 | 60.9 ± 7 | 60 ± 6.6 | | 27.42 ± 6.89 | 28.24 ± 8.77 | | NR | NR | | 7.5 (3-10)* | 7.0 (5-10)* |
| Paterson 2016 [36] | HA | 10 | | 9 | 19 | 8/2 | 7/2 | 49.91 ± 13.72 | 52.70 ± 10.30 | | 27.92 ± 11.94 | 30.87 ± 5.64 | | NR | NR | | 48.09 ± 23.75 | 39.70 ± 21.90 |
| Filardo 2015 [37] | HA | 94 | | 89 | 183 | 60/34 | 52/37 | 53.32  ± 13.2 | 57.55  ± 11.8 | | 26.6 ± 4.0 | 26.9 ± 4.4 | | NR | NR | | 73.2 ± 12.0 | 71.6 ±13.4 |
| Raeissadat 2014-15 [38] | HA | 77 | | 62 | 139 | 8/69 | 15/47 | 56.85 ± 9.13 | 61.13 ± 7.48 | | 28.20 ± 4.63 | 27.03 ± 4.15 | | 39.5 ± 17.06 | 28.69 ± 16.69 | | NR | NR |
| Patel 2013 [39] | Saline | single PRP: 26 patients/ 52 knees. Double PRP: 25 patients/50 knees | | 23 patients/46 knees | 74 Patients/148 knees | Single PRP: 11/16 Double PRP: 5/20 | 6/17 | Single PRP: 53.11 ± 11.55 Double PRP:  51.64 ± 9.22 | 53.65 ± 8.17 | | Single PRP: 26.28 ± 3.23 Double PRP: 25.81 ± 3.31 | 26.21 ± 2.93 | | Single PRP: 49.56 ± 17.83  Double PRP: 53.20 ± 16.18 | 45.54 ± 17.29 | | Single PRP: 4.56 ± 0.61  Double PRP: 4.64 ± 0.56 | 4.57 ± 0.62 |
| Vaquerizo 2013 [40] | HA | 48 | | 42 | 90 | 16/32 | 16/26 | 62.4 ± 6.6 | 64.8 ± 7.7 | | 30.7± 3.6 | 31.0 ±4.6 | | 45.9 ± 12.7 | 50.8 ± 18.4 | | NR | NR |
| Cerza 2012 [41] | HA | Left knee:17 Right knee:43 | | Left Knee:12  Right Knee:48 | 120 | 25/35 | 28/32 | 66.5 ± 11.3 | 66.2 ± 10.6 | | NR | NR | | 76.9 ± 9.5 | 75.4 ± 10.7 | | NR | NR |
| Filardo 2012 [42] | HA | 54 | | 55 | 109 | 37/17 | 31/24 | 55 | 58 | | 27 | 26 | | NR | NR | | NR | NR |

**Table S1: Baseline characteristics of include participants**

**Abbreviations:** PRP = Platelet Rich Plasma; HA = Hyaluronic acid; CS = corticosteroid; PRL = prolotherapy; BMI = Body mass Index; WOMAC = Western Ontario and McMaster Universities Arthritis Index; VAS = Visual Analog Scale.

| Article | Selection Bias | | Performance Bias | Detection bias | Attrition bias | Reporting Bias | Other bias | Our evaluation |
| --- | --- | --- | --- | --- | --- | --- | --- | --- |
|  | **Random**  **Sequence**  **Generation** | **Allocation concealment** | **Blinding of participants and personnel** | **Blinding of outcome assessment** | **Incomplete outcome data** | **Selective Reporting** | **Anything**  **else, ideally prespecified** |  |
| Ying-Chun Wang [1] | Low risk | Low risk | Low risk | Low risk | Low risk | Low risk | Low risk | Good |
| Fatima Nur Kesiktas [2] | Low risk | Unclear risk | High risk | Low risk | Low risk | Low risk | Low risk | Fair |
| Hung-  Ya  Huang [3] | Low risk | Low risk | Low risk | Low risk | Low risk | Low risk | Unclear risk | Good |
| Andrejs  Elksniņš-Finogejev s [4] | Low risk | Unclear risk | High risk | Low risk | High risk | Low risk | High risk | Poor |
| Kim L Bennell [5] | Low risk | Low risk | Low risk | Low risk | Low risk | Low risk | Low risk | Good |
| Seyed Ahmad Raeissadat [6] | Low risk | Low risk | Low risk | Low risk | Low risk | Low risk | Low risk | Good |
| Yong-Beom Park [7] | Low risk | Low risk | Low risk | Low risk | Low risk | Low risk | Low risk | Good |
| Oliver Dulic [8] | Low risk | Unclear risk | High risk | High risk | Low risk | Low risk | Low risk | Poor |
| Murillo Dorio [9] | Low risk | Low risk | Low risk | Low risk | Low risk | Low risk | Low risk | Good |
| Jason D. Tucker [10] | Low risk | Unclear risk | Low risk | High risk | Low risk | Low risk | Low risk | Fair |
| Medhat SDEEK [11] | Low risk | Low risk | Low risk | Low risk | Low risk | Low risk | Low risk | Good |
| Huseyin Elik [12] | Low Risk | Low Risk | Low Risk | Low Risk | Low Risk | Low Risk | Low Risk | Good |
| Marianna Ribeiro de Menezes Freire [13] | Low Risk | Low Risk | Low Risk | Low Risk | Low Risk | Unclear Risk | Low Risk | Good |
| Alessandro Di Martino [14] | Low risk | Low risk | Low risk | Low risk | Low risk | Low risk | Low risk | Good |
| Nahla M. Gaballa [15] | Low Risk | Unclear Risk | High Risk | High Risk | Unclear Risk | Low Risk | Low Risk | Fair |
| Babita Ghai [16] | Low Risk | Low Risk | Low Risk | Low Risk | Low Risk | Low Risk | Low Risk | Good |
| Yong Huang [17] | Low Risk | Unclear Risk | High Risk | High Risk | Low Risk | Low Risk | Low Risk | Poor |
| Poupak Rahimzadeh [18] | Low Risk | Low Risk | Low Risk | Low Risk | Low Risk | Unclear Risk | Low Risk | Good |
| Mikel Sanchez [19] | Low Risk | Low Risk | Low Risk | Low Risk | Low Risk | Low Risk | Low Risk | Good |
| Ece Uslu Guvendi [20] | Low Risk | Low Risk | High Risk | Low Risk | Low Risk | Low Risk | Low Risk | Fair |
| Bahram Naderi Nabi [21] | Low Risk | Low Risk | High Risk | Low Risk | Unclear Risk | Unclear Risk | Low Risk | Poor |
| Marie Laure Louis [22] | Low Risk | Low Risk | Low Risk | Low Risk | Low Risk | Low Risk | Low Risk | Good |
| Hamada S. Ahmad [23] | Low Risk | Unclear Risk | High Risk | Low Risk | Unclear Risk | Low Risk | Low Risk | Poor |
| David  Buendía-  López [24] | Low risk | Unclear risk | High risk | Low risk | Low risk | Low risk | Low risk | Fair |
| Ke Su [25] | Low risk | Unclear risk | High risk | High risk | Low risk | Low risk | Low risk | Poor |
| Tahir Mutlu Duymus [26] | Low risk | High risk | High risk | High risk | Low risk | Low risk | Low risk | Poor |
| Nayana Joshi Jubert [27] | Low risk | Low risk | Low risk | Low risk | Low risk | Low risk | High risk | Fair |
| Brian J. Cole [28] | Low risk | Unclear risk | Low risk | Low risk | Low risk | Low risk | Low risk | Good |
| Gokay Gormeli [29] | Low risk | Unclear risk | Low risk | Low risk | Low risk | Low risk | Low risk | Good |
| Kuan-Yu Lin [30] | Low Risk | Low Risk | Low Risk | Low Risk | Low Risk | Low Risk | Low Risk | Good |
| Claudio Lisi [31] | Low Risk | Low Risk | Low Risk | Low Risk | Low Risk | Unclear Risk | Low Risk | Good |
| Seyed Ahmad Raeissadat – 2017 [32] | Low Risk | Low Risk | High Risk | Low Risk | Low Risk | Unclear Risk | Low Risk | Fair |
| Patrick A. Smith [33] | Low risk | Low risk | Low risk | Low risk | Low risk | Low risk | Low risk | Good |
| Elvira Montanez-Heredia [34] | Low risk | Low risk | Low risk | Low risk | Low risk | Low risk | Low risk | Good |
| Lana JFSD [35] | Low Risk | Low Risk | Low Risk | Low Risk | Low Risk | Unclear Risk | Low Risk | Good |
| Kade L. Paterson [36] | Low Risk | Low Risk | Low Risk | Low Risk | Low Risk | Low Risk | Low Risk | Good |
| Giuseppe Filardo [37] | Unclear risk | Low risk | Low risk | Low risk | Low risk | Low risk | Low risk | Good |
| Seyed Ahmad Raeissadat – 2014 [38] | Low Risk | Low Risk | High Risk | High Risk | Low Risk | Unclear Risk | Low Risk | Poor |
| Sandeep Patel [39] | Low Risk | Low Risk | Low Risk | Low Risk | High Risk | Low Risk | Low Risk | Fair |
| Victor Vaquerizo [40] | Low Risk | Low Risk | Low risk | Low Risk | Low Risk | Low Risk | Low Risk | Good |
| Cerza [41] | Low risk | Unclear risk | High risk | Low risk | Low risk | Low risk | Low risk | Fair |
| Giuseppe Filardo [42] | Low Risk | Low Risk | Low Risk | Low Risk | Low Risk | Low Risk | Low Risk | Good |

**Table S2: Quality assessment of Randomized Controlled Trials by Cochrane Risk of bias tool.**

| Outcome | t value | df | Lower CI | Upper CI | P value |
| --- | --- | --- | --- | --- | --- |
| PRP vs HA |  |  |  |  |  |
| WOMAC total | 5.807 | 32 | -15.222 | -7.317 | 0.00000 |
| WOMAC pain | 2.402 | 26 | -12.799 | -0.995 | 0.02372 |
| WOMAC stiffness | 2.184 | 22 | -10.115 | -0.262 | 0.03988 |
| WOMAC function | 5.818 | 20 | -16.338 | -7.771 | 0.00001 |
| VAS pain | 3.820 | 20 | -12.230 | -3.591 | 0.00107 |
| IKDC score | 4.043 | 19 | 1.735 | 5.459 | 0.00069 |
| PRP vs CS |  |  |  |  |  |
| WOMAC total | 1.910 | 2 | -39.423 | 15.177 | 0.19623 |
| WOMAC pain | 1.804 | 1 | -75.432 | 56.674 | 0.32220 |
| VAS pain | 2.515 | 9 | -12.368 | -0.655 | 0.03302 |
| KOOS pain | 3.672 | 4 | 9.858 | 70.904 | 0.02134 |
| PRP vs Placebo |  |  |  |  |  |
| WOMAC total | 4.724 | 21 | -22.399 | -8.706 | 0.00012 |
| WOMAC pain | 3.533 | 14 | -18.718 | -4.5773 | 0.00331 |
| WOMAC stiffness | 3.025 | 14 | -20.600 | -3.509 | 0.00908 |
| WOMAC function | 2.891 | 14 | -21.685 | -3.214 | 0.01185 |
| VAS pain | 1.814 | 7 | -31.614 | 4.157 | 0.11239 |

**Table S3: Results of Eggers test for publication bias**

**Abbreviations:** PRP = Platelet Rich Plasma; HA = Hyaluronic acid; CS = corticosteroid; WOMAC = Western Ontario and McMaster Universities Arthritis Index; VAS = Visual Analog Scale; KOOS = Knee Injury and Osteoarthritis Outcome Score; IKDC = International Knee Documentation Committee

**REFERENCES:**

1. Wang Y-C, Lee C-L, Chen Y-J, Tien Y-C, Lin S-Y, Chen C-H, Chou PP-H, Huang H-T. Comparing the Efficacy of Intra-Articular Single Platelet-Rich Plasma(PRP) versus Novel Crosslinked Hyaluronic Acid for Early-Stage Knee Osteoarthritis: A Prospective, Double-Blind, Randomized Controlled Trial. Medicina. 2022; 58(8):1028. <https://doi.org/10.3390/medicina58081028>
2. Kesiktas, F.N., Dernek, B., Sen, E.I. *et al.* Comparison of the short-term results of single-dose intra-articular peptide with hyaluronic acid and platelet-rich plasma injections in knee osteoarthritis: a randomized study. *Clin Rheumatol* **39**, 3057–3064 (2020). <https://doi.org/10.1007/s10067-020-05121-4>
3. Huang, HY., Hsu, CW., Lin, GC. *et al.* Comparing efficacy of a single intraarticular injection of platelet-rich plasma (PRP) combined with different hyaluronans for knee osteoarthritis: a randomized-controlled clinical trial. *BMC Musculoskelet Disord* **23**, 954 (2022). <https://doi.org/10.1186/s12891-022-05906-5>
4. Elksniņš-Finogejevs, A., Vidal, L. & Peredistijs, A. Intra-articular platelet-rich plasma vs corticosteroids in the treatment of moderate knee osteoarthritis: a single-center prospective randomized controlled study with a 1-year follow up. *J Orthop Surg Res* **15**, 257 (2020). <https://doi.org/10.1186/s13018-020-01753-z>
5. Bennell KL, Paterson KL, Metcalf BR, et al. Effect of Intra-articular Platelet-Rich Plasma vs Placebo Injection on Pain and Medial Tibial Cartilage Volume in Patients With Knee Osteoarthritis: The RESTORE Randomized Clinical Trial. JAMA. 2021;326(20):2021–2030. doi:10.1001/jama.2021.19415
6. Raeissadat, S.A., Ghazi Hosseini, P., Bahrami, M.H. *et al.* The comparison effects of intra-articular injection of Platelet Rich Plasma (PRP), Plasma Rich in Growth Factor (PRGF), Hyaluronic Acid (HA), and ozone in knee osteoarthritis; a one year randomized clinical trial. *BMC Musculoskelet Disord* **22**, 134 (2021). <https://doi.org/10.1186/s12891-021-04017-x>
7. Park YB, Kim JH, Ha CW, Lee DH. Clinical Efficacy of Platelet-Rich Plasma Injection and Its Association With Growth Factors in the Treatment of Mild to Moderate Knee Osteoarthritis: A Randomized Double-Blind Controlled Clinical Trial As Compared With Hyaluronic Acid. Am J Sports Med. 2021 Feb;49(2):487-496. doi: 10.1177/0363546520986867. PMID: 33523756.
8. Dulic O, Rasovic P, Lalic I, Kecojevic V, Gavrilovic G, Abazovic D, Maric D, Miskulin M, Bumbasirevic M. Bone Marrow Aspirate Concentrate versus Platelet Rich Plasma or Hyaluronic Acid for the Treatment of Knee Osteoarthritis. Medicina. 2021; 57(11):1193. <https://doi.org/10.3390/medicina57111193>
9. Dório, M., Pereira, R.M.R., Luz, A.G.B. *et al.* Efficacy of platelet-rich plasma and plasma for symptomatic treatment of knee osteoarthritis: a double-blinded placebo-controlled randomized clinical trial. *BMC Musculoskelet Disord* **22**, 822 (2021). <https://doi.org/10.1186/s12891-021-04706-7>
10. Tucker JD, Goetz LL, Duncan MB, Gilman JB, Elmore LW, Sell SA, McClure MJ, Quagliano PV, Martin CC. Randomized, Placebo‐Controlled Analysis of the Knee Synovial Environment Following Platelet‐Rich Plasma Treatment for Knee Osteoarthritis. PM&R. 2021 Jul;13(7):707-19.
11. Sdeek M, Sabry D, El-Sdeek H, Darweash A. Intra-articular injection of Platelet rich plasma versus Hyaluronic acid for moderate knee osteoarthritis. A prospective, double-blind randomized controlled trial on 189 patients with follow-up for three years. Acta Orthop Belg. 2021 Dec 1;87(4):729-34.
12. Elik H, Doğu B, Yılmaz F, Begoğlu FA, Kuran B. The efficiency of platelet-rich plasma treatment in patients with knee osteoarthritis. J Back Musculoskelet Rehabil. 2020;33(1):127-138. doi: 10.3233/BMR-181374. PMID: 31127755.
13. Freire MR, da Silva PM, Azevedo AR, Silva DS, da Silva RB, Cardoso JC. Comparative effect between infiltration of platelet-rich plasma and the use of corticosteroids in the treatment of knee osteoarthritis: a prospective and randomized clinical trial. Revista Brasileira de Ortopedia. 2020 Dec 2;55:551-6.
14. Di Martino A, Di Matteo B, Papio T, Tentoni F, Selleri F, Cenacchi A, Kon E, Filardo G. Platelet-rich plasma versus hyaluronic acid injections for the treatment of knee osteoarthritis: results at 5 years of a double-blind, randomized controlled trial. The American Journal of Sports Medicine. 2019 Feb;47(2):347-54.
15. Gaballa NM, Mohammed YA, Kamel LM, Mahgoub HM. Therapeutic efficacy of intra-articular injection of platelet–rich plasma and ozone therapy in patients with primary knee osteoarthritis. The Egyptian Rheumatologist. 2019 Jul 1;41(3):183-7.
16. Ghai B, Gupta V, Jain A, Goel N, Chouhan D, Batra YK. Effectiveness of platelet rich plasma in pain management of osteoarthritis knee: double blind, randomized comparative study. Revista Brasileira de Anestesiologia. 2019 Dec 20;69:439-47.
17. Huang, Y., Liu, X., Xu, X. *et al.* Intra-articular injections of platelet-rich plasma, hyaluronic acid or corticosteroids for knee osteoarthritis. *Orthopäde* **48**, 239–247 (2019). <https://doi.org/10.1007/s00132-018-03659-5>
18. Rahimzadeh P, Imani F, Faiz SH, Entezary SR, Zamanabadi MN, Alebouyeh MR. The effects of injecting intra-articular platelet-rich plasma or prolotherapy on pain score and function in knee osteoarthritis. Clinical Interventions in Aging. 2018 Jan 4:73-9.
19. Sánchez M, Fiz N, Azofra J, Usabiaga J, Recalde EA, Gutierrez AG, Albillos J, Gárate R, Aguirre JJ, Padilla S, Orive G. A randomized clinical trial evaluating plasma rich in growth factors (PRGF-Endoret) versus hyaluronic acid in the short-term treatment of symptomatic knee osteoarthritis. Arthroscopy: The Journal of Arthroscopic & Related Surgery. 2012 Aug 1;28(8):1070-8.
20. Uslu Güvendi E, Aşkin A, Güvendi G, Koçyiğit H. Comparison of Efficiency Between Corticosteroid and Platelet Rich Plasma Injection Therapies in Patients With Knee Osteoarthritis. Arch Rheumatol. 2017 Nov 2;33(3):273-281. doi: 10.5606/ArchRheumatol.2018.6608. PMID: 30632533; PMCID: PMC6328223.
21. Nabi BN, Sedighinejad A, Mardani-Kivi M, Haghighi M, Roushan ZA, Biazar G. Comparing the effectiveness of intra-articular platelet-rich plasma and corticosteroid injection under ultrasound guidance on pain control of knee osteoarthritis. Iranian Red Crescent Medical Journal. 2018 Mar 1;20(3).
22. Louis, M. L., Magalon, J., Jouve, E., Bornet, C. E., Mattei, J. C., Chagnaud, C., … Sabatier, F. (2018). *Growth Factors Levels Determine Efficacy of Platelets Rich Plasma Injection in Knee Osteoarthritis: A Randomized Double Blind Noninferiority Trial Compared With Viscosupplementation. Arthroscopy: The Journal of Arthroscopic & Related Surgery, 34(5), 1530–1540.e2.* doi:10.1016/j.arthro.2017.11.035
23. Ahmad HS, Farrag SE, Okasha AE, Kadry AO, Ata TB, Monir AA, Shady I. Clinical outcomes are associated with changes in ultrasonographic structural appearance after platelet‐rich plasma treatment for knee osteoarthritis. International journal of rheumatic diseases. 2018 May;21(5):960-6.
24. Buendía-López, D., Medina-Quirós, M. & Fernández-Villacañas Marín, M. Clinical and radiographic comparison of a single LP-PRP injection, a single hyaluronic acid injection and daily NSAID administration with a 52-week follow-up: a randomized controlled trial. *J Orthop Traumatol* **19**, 3 (2018). <https://doi.org/10.1186/s10195-018-0501-3>
25. Su, K., Bai, Y., Wang, J. *et al.* Comparison of hyaluronic acid and PRP intra-articular injection with combined intra-articular and intraosseous PRP injections to treat patients with knee osteoarthritis. *Clin Rheumatol* **37**, 1341–1350 (2018). <https://doi.org/10.1007/s10067-018-3985-6>
26. Duymus TM, Mutlu S, Dernek B, Komur B, Aydogmus S, Kesiktas FN. Choice of intra-articular injection in treatment of knee osteoarthritis: platelet-rich plasma, hyaluronic acid or ozone options. Knee surgery, sports traumatology, arthroscopy. 2017 Feb;25:485-92.
27. Joshi Jubert N, Rodríguez L, Reverté-Vinaixa MM, Navarro A. Platelet-rich plasma injections for advanced knee osteoarthritis: a prospective, randomized, double-blinded clinical trial. Orthopaedic journal of sports medicine. 2017 Feb 13;5(2):2325967116689386.
28. Cole BJ, Karas V, Hussey K, Merkow DB, Pilz K, Fortier LA. Hyaluronic Acid Versus Platelet-Rich Plasma: A Prospective, Double-Blind Randomized Controlled Trial Comparing Clinical Outcomes and Effects on Intra-articular Biology for the Treatment of Knee Osteoarthritis. *The American Journal of Sports Medicine*. 2017;45(2):339-346. doi:[10.1177/0363546516665809](https://doi.org/10.1177/0363546516665809)
29. Görmeli G, Görmeli CA, Ataoglu B, Çolak C, Aslantürk O, Ertem K. Multiple PRP injections are more effective than single injections and hyaluronic acid in knees with early osteoarthritis: a randomized, double-blind, placebo-controlled trial. Knee Surgery, Sports Traumatology, Arthroscopy. 2017 Mar;25(3):958-65.
30. Lin, K.-Y., Yang, C.-C., Hsu, C.-J., Yeh, M.-L., & Renn, J.-H. (2019). *Intra-articular Injection of Platelet-Rich Plasma Is Superior to Hyaluronic Acid or Saline Solution in the Treatment of Mild to Moderate Knee Osteoarthritis: A Randomized, Double-Blind, Triple-Parallel, Placebo-Controlled Clinical Trial. Arthroscopy: The Journal of Arthroscopic & Related Surgery, 35(1), 106–117.* doi:10.1016/j.arthro.2018.06.035
31. Lisi C, Perotti C, Scudeller L, Sammarchi L, Dametti F, Musella V, Di Natali G. Treatment of knee osteoarthritis: platelet-derived growth factors vs. hyaluronic acid. A randomized controlled trial. Clinical Rehabilitation. 2018 Mar;32(3):330-9.
32. Raeissadat SA, Rayegani SM, Ahangar AG, Abadi PH, Mojgani P, Ahangar OG. Efficacy of Intra-articular Injection of a Newly Developed Plasma Rich in Growth Factor (PRGF) Versus Hyaluronic Acid on Pain and Function of Patients with Knee Osteoarthritis: A Single-Blinded Randomized Clinical Trial. *Clinical Medicine Insights: Arthritis and Musculoskeletal Disorders*. 2017;10. doi:[10.1177/1179544117733452](https://doi.org/10.1177/1179544117733452)
33. Smith PA. Intra-articular Autologous Conditioned Plasma Injections Provide Safe and Efficacious Treatment for Knee Osteoarthritis: An FDA-Sanctioned, Randomized, Double-blind, Placebo-controlled Clinical Trial. *The American Journal of Sports Medicine*. 2016;44(4):884-891. doi:[10.1177/0363546515624678](https://doi.org/10.1177/0363546515624678)
34. Montañez-Heredia E, Irízar S, Huertas PJ, Otero E, Del Valle M, Prat I, Díaz-Gallardo MS, Perán M, Marchal JA, Hernandez-Lamas MD. Intra-articular injections of platelet-rich plasma versus hyaluronic acid in the treatment of osteoarthritic knee pain: a randomized clinical trial in the context of the Spanish National Health Care System. International Journal of Molecular Sciences. 2016 Jul 2;17(7):1064.
35. Lana JF, Weglein A, Sampson SE, Vicente EF, Huber SC, Souza CV, Ambach MA, Vincent H, Urban-Paffaro A, Onodera CM, Annichino-Bizzacchi JM. Randomized controlled trial comparing hyaluronic acid, platelet-rich plasma and the combination of both in the treatment of mild and moderate osteoarthritis of the knee. Journal of stem cells & regenerative medicine. 2016;12(2):69.
36. Paterson KL, Nicholls M, Bennell KL, Bates D. Intra-articular injection of photo-activated platelet-rich plasma in patients with knee osteoarthritis: a double-blind, randomized controlled pilot study. BMC Musculoskeletal Disorders. 2016 Dec;17:1-9.
37. Filardo G, Di Matteo B, Di Martino A, et al. Platelet-Rich Plasma Intra-articular Knee Injections Show No Superiority Versus Viscosupplementation: A Randomized Controlled Trial. *The American Journal of Sports Medicine*. 2015;43(7):1575-1582. doi:[10.1177/0363546515582027](https://doi.org/10.1177/0363546515582027)
38. Raeissadat SA, Rayegani SM, Hassanabadi H, et al. Knee Osteoarthritis Injection Choices: Platelet- Rich Plasma (PRP) versus Hyaluronic Acid (A one-year randomized clinical trial). *Clinical Medicine Insights: Arthritis and Musculoskeletal Disorders*. 2015;8. doi:[10.4137/CMAMD.S17894](https://doi.org/10.4137/CMAMD.S17894)
39. Patel S, Dhillon MS, Aggarwal S, Marwaha N, Jain A. Treatment with platelet-rich plasma is more effective than placebo for knee osteoarthritis: a prospective, double-blind, randomized trial. Am J Sports Med. 2013 Feb;41(2):356-64. doi: 10.1177/0363546512471299. Epub 2013 Jan 8. PMID: 23299850.
40. Vaquerizo V, Plasencia MÁ, Arribas I, Seijas R, Padilla S, Orive G, Anitua E. Comparison of intra-articular injections of plasma rich in growth factors (PRGF-Endoret) versus Durolane hyaluronic acid in the treatment of patients with symptomatic osteoarthritis: a randomized controlled trial. Arthroscopy: The Journal of Arthroscopic & Related Surgery. 2013 Oct 1;29(10):1635-43.
41. Cerza F, Carnì S, Carcangiu A, Di Vavo I, Schiavilla V, Pecora A, De Biasi G, Ciuffreda M. Comparison between hyaluronic acid and platelet-rich plasma, intra-articular infiltration in the treatment of gonarthrosis. The American journal of sports medicine. 2012 Dec;40(12):2822-7.
42. Filardo, G., Kon, E., Di Martino, A. *et al.* Platelet-rich plasma vs hyaluronic acid to treat knee degenerative pathology: study design and preliminary results of a randomized controlled trial. *BMC Musculoskelet Disord* **13**, 229 (2012). <https://doi.org/10.1186/1471-2474-13-229>
