# Supplementary material for: Comparative effectiveness of intra-articular therapies in knee osteoarthritis: a meta-analysis comparing platelet-rich plasma (PRP) with other treatment modalities
Source: Ann Med Surg (Lond). 2023 Dec 15;86(1):361–72. doi: 10.1097/MS9.0000000000001615 (PMC10783230; doi:10.1097/MS9.0000000000001615)
Supplement: SUPPLEMENTARY MATERIAL [file ms9-86-361-s004.docx]

**SUPPLEMENTARY FIGURES**

**Funnel plots**

A)
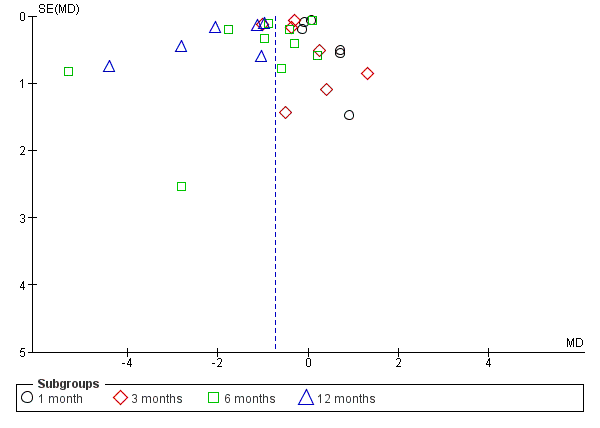
B)
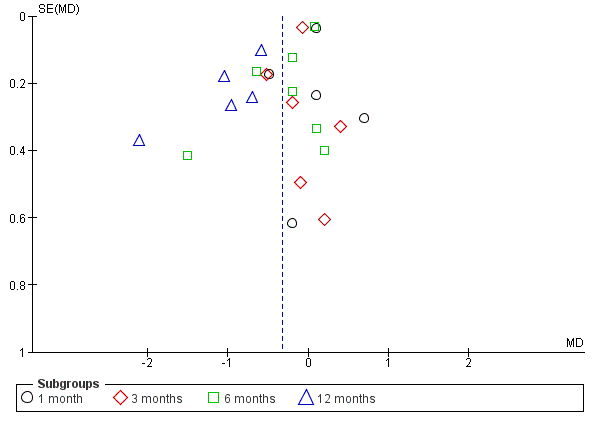


C)
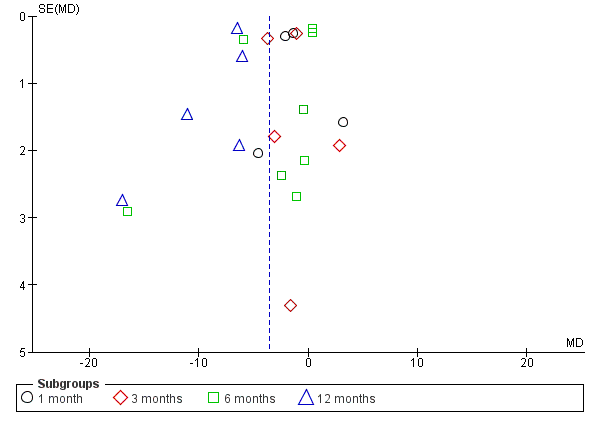
D)
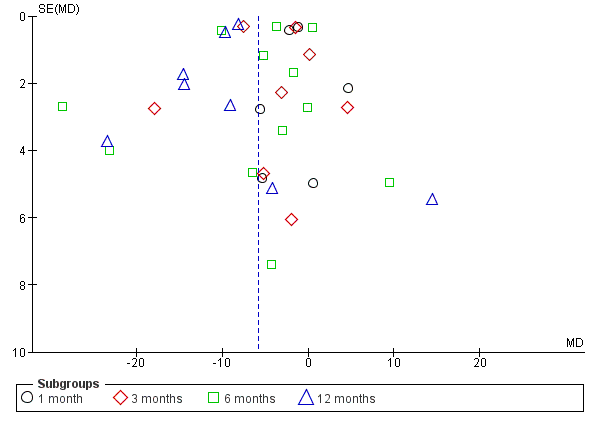


E)
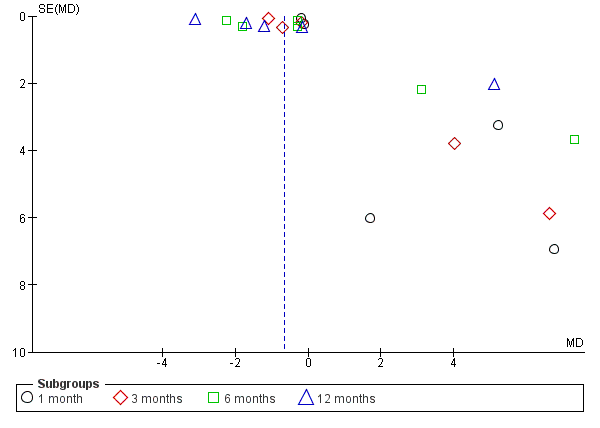
F)
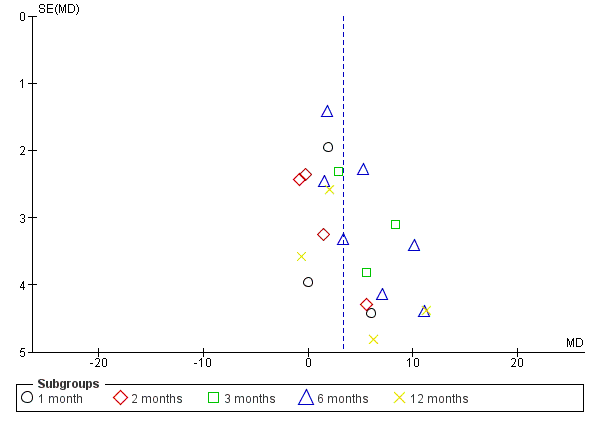


**Figure S1:** Funnel plots for the subgroup Platelet rich plasma (PRP) Vs Hyaluronic acid (HA) for the outcomes of (A) WOMAC pain (B) WOMAC stiffness (C) WOMAC function (D) WOMAC total (E) VAS pain (F) IKDC at 1, 3, 6 and 12 months.

A)
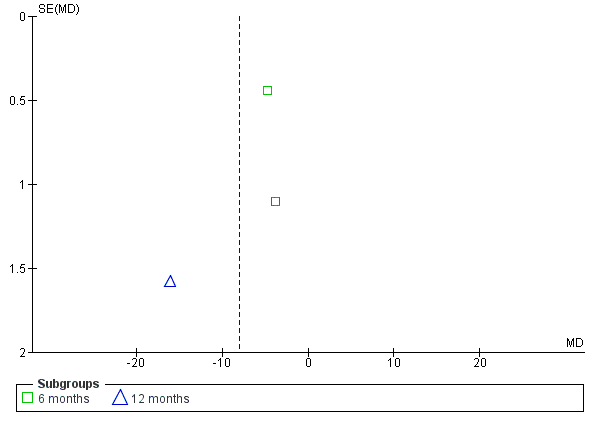
B)
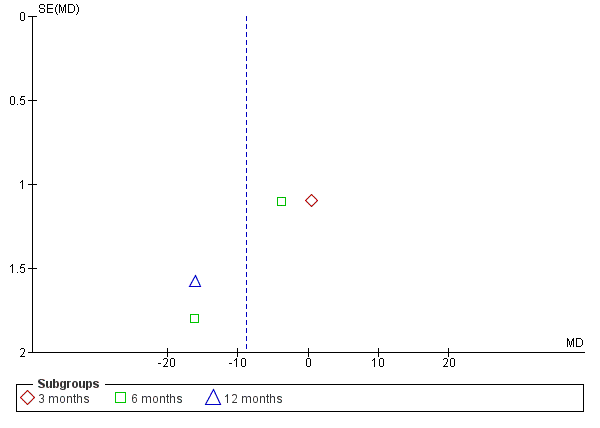


C)
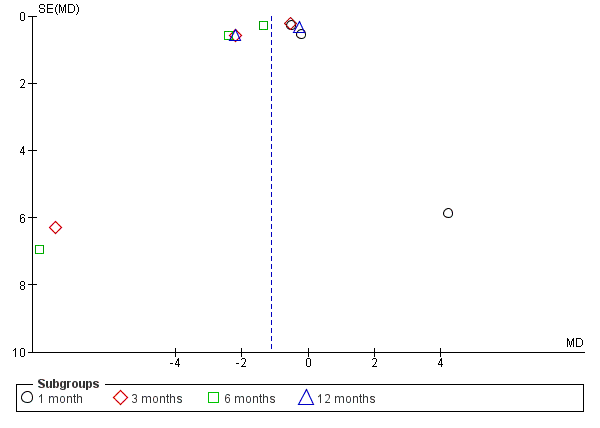
D)
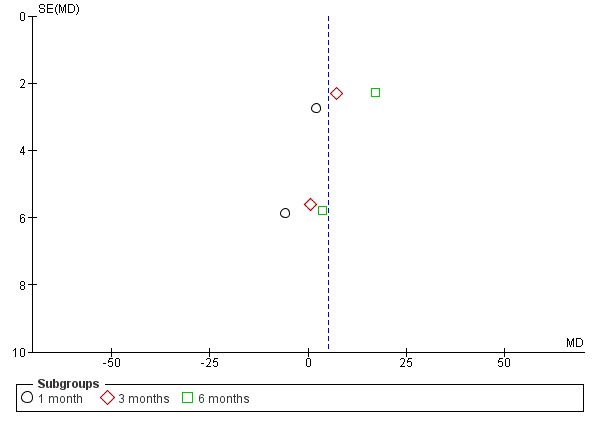


**Figure S2**: Funnel plots for the subgroup Platelet rich plasma (PRP) Vs Steroids for the outcomes of (A) WOMAC pain (B) WOMAC total (C) VAS pain (D) KOOS pain at 1, 3, 6, and 12 months.

A
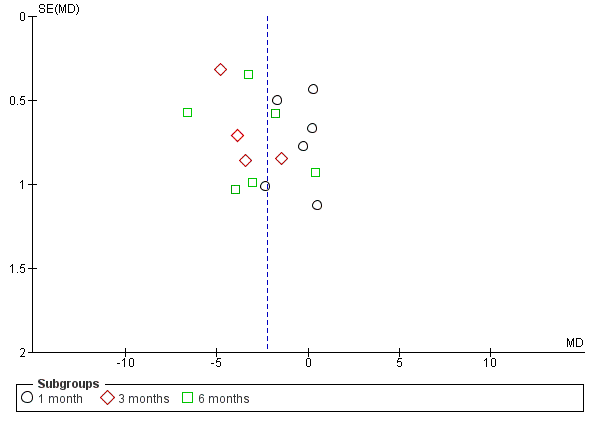
B
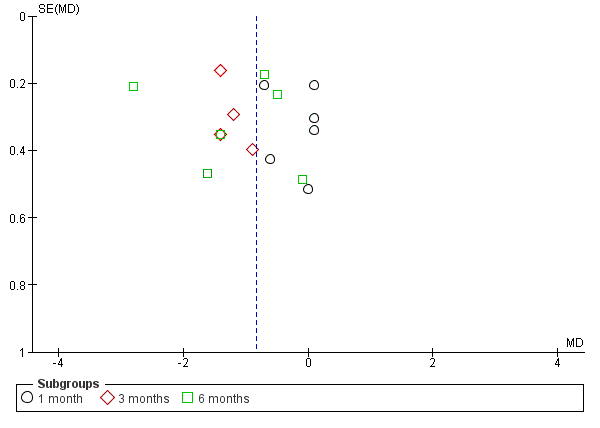


C
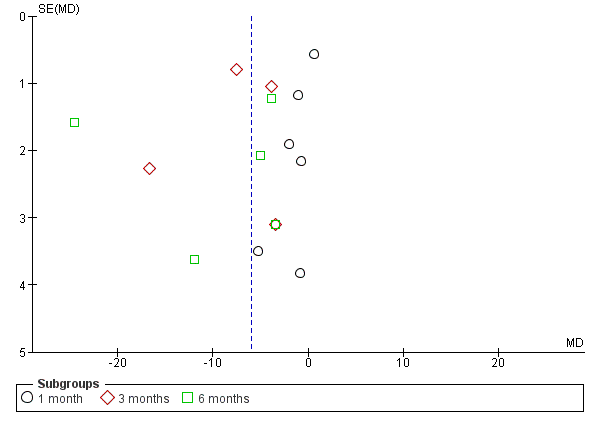
D
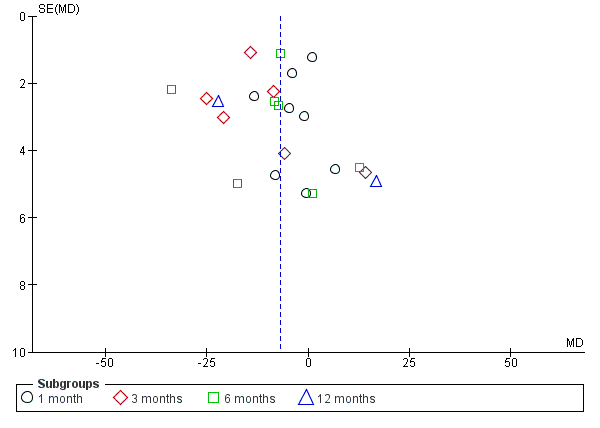


E
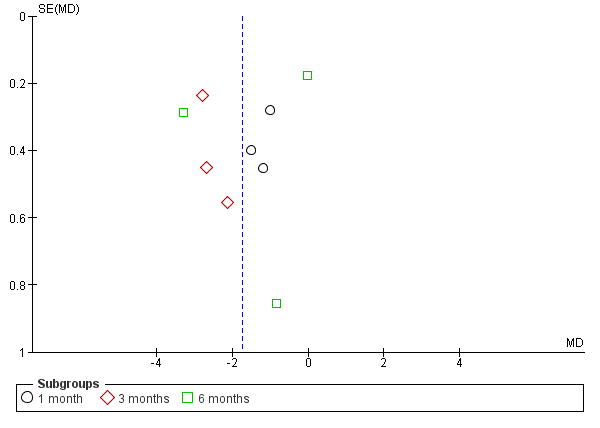


**Figure S3:** Funnel plots for the subgroup Platelet rich plasma (PRP) Vs Placebo for the outcomes of (A) WOMAC pain (B) WOMAC stiffness (C) WOMAC function (D) WOMAC total (E) VAS pain at 1, 3, 6, and 12 months

**Sensitivity Analysis and Forest Plots**

**
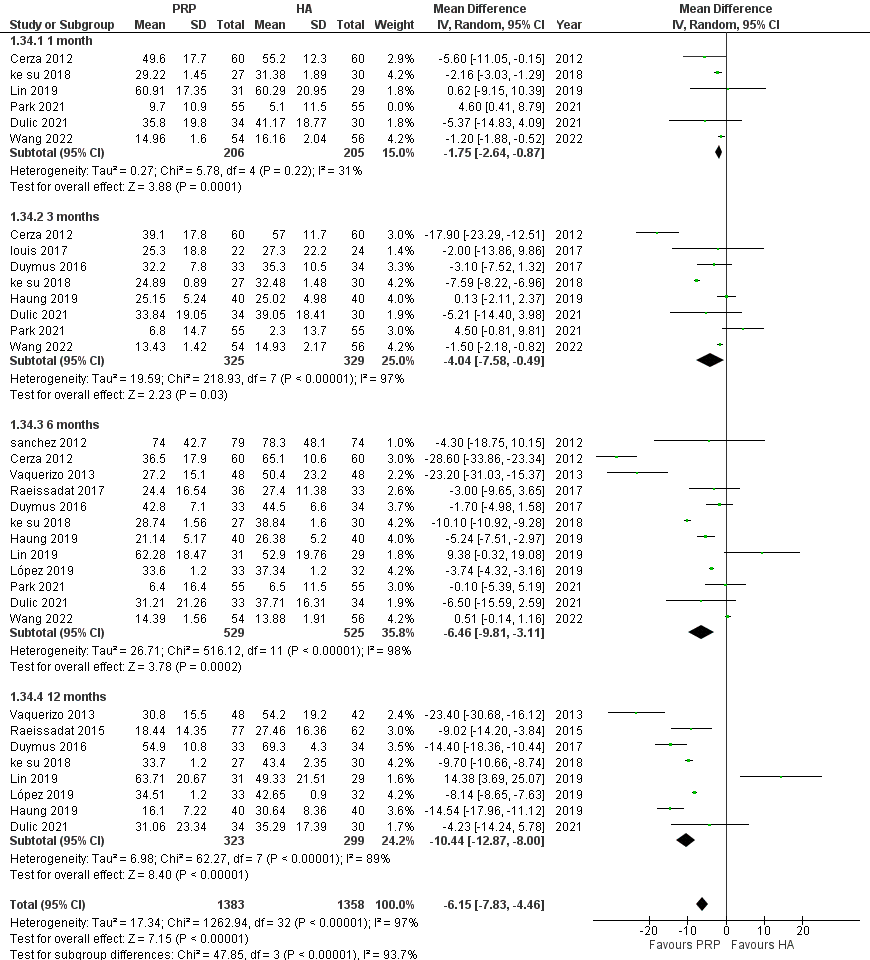
**

**Figure S4:** Sensitivity analysis for the subgroup Platelet rich plasma (PRP) vs Hyaluronic acid (HA) for the outcome of WOMAC total at 1 month

v**
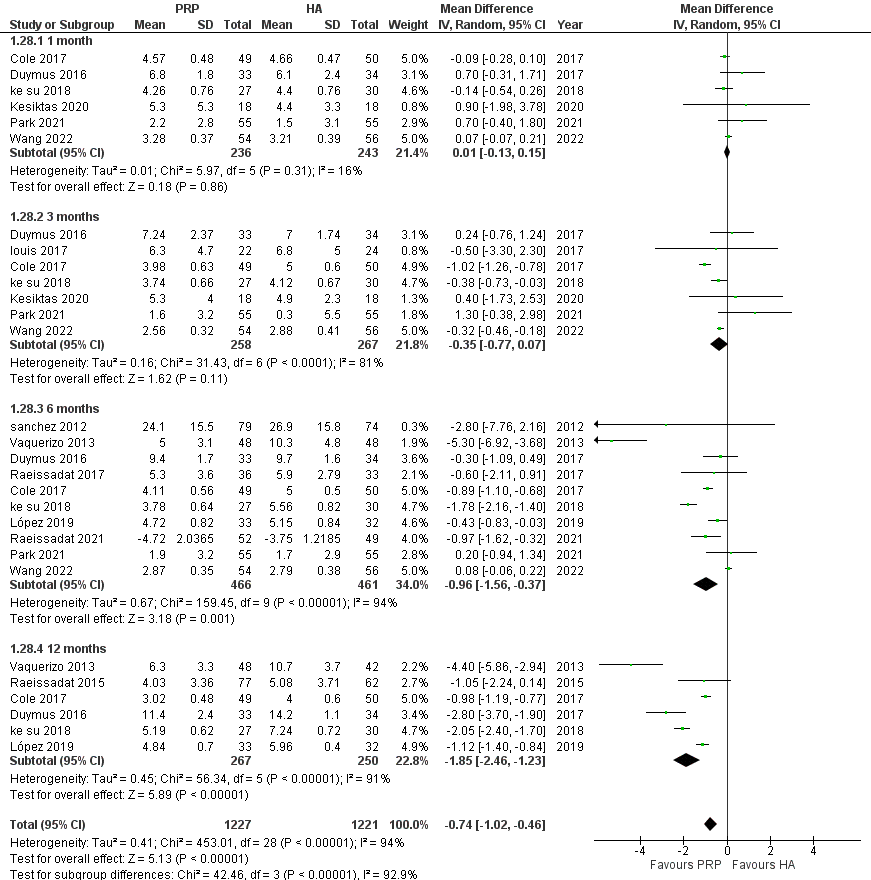
**

**Figure S5:** Forest plots for the subgroup analysis of Platelet rich plasma (PRP) Vs Hyaluronic acid (HA) for the outcome of WOMAC pain at 1, 3, 6, and 12 months

**
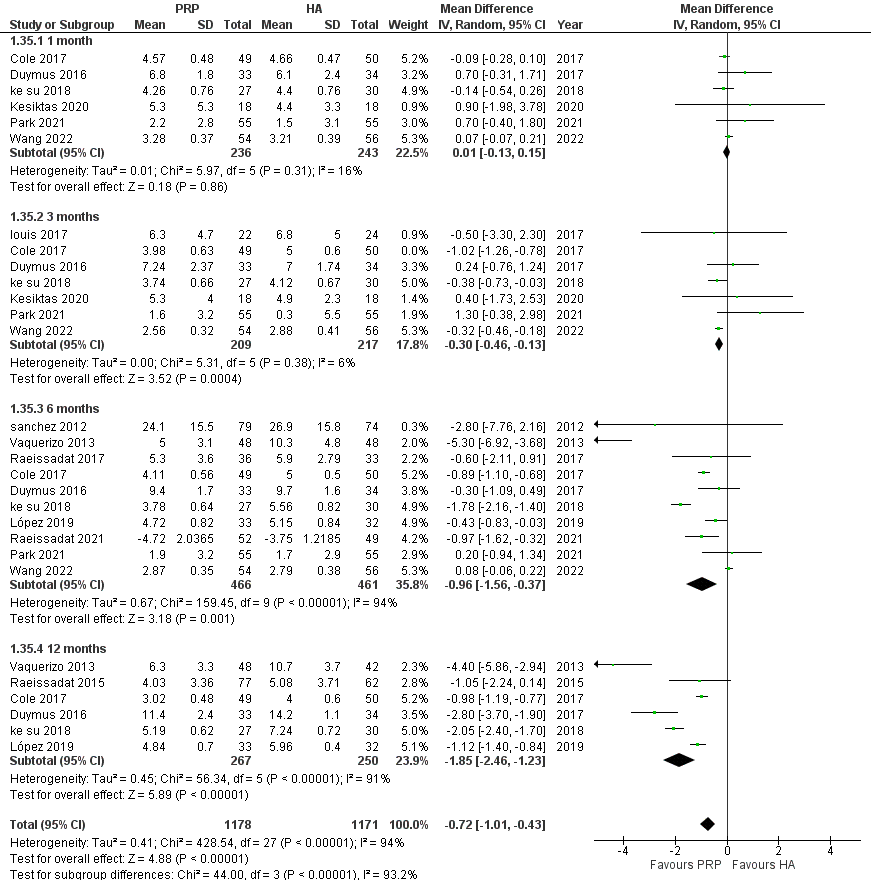
**

**Figure S6:** Sensitivity analysis for the subgroup Platelet rich plasma (PRP) vs Hyaluronic acid (HA) for the outcome of WOMAC pain at 3 months

**
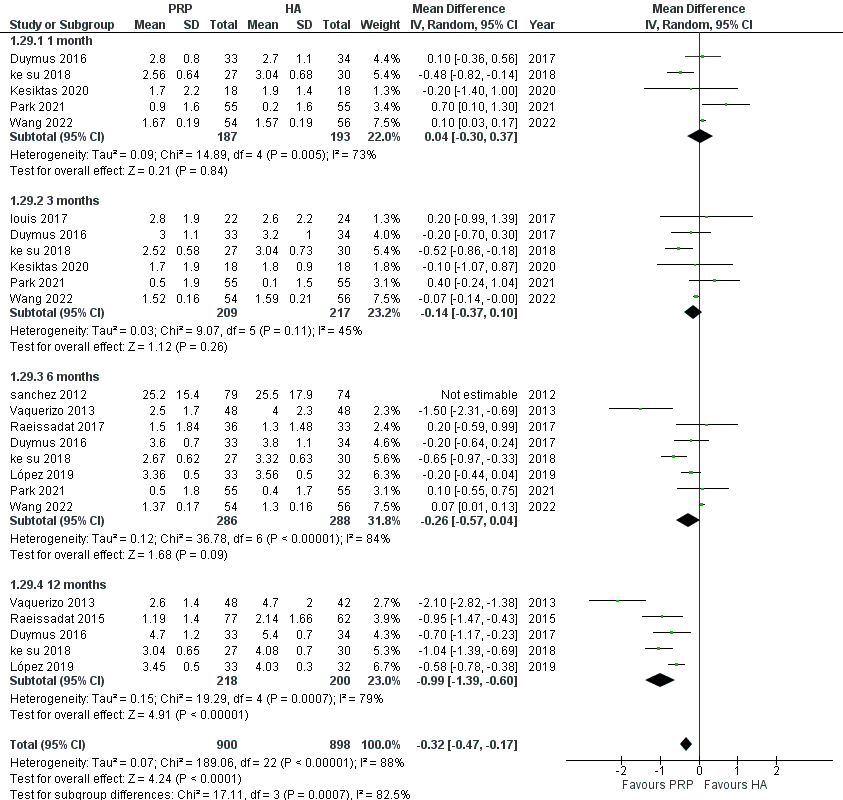
**

**Figure S7:** Forest plots for the subgroup analysis of Platelet rich plasma (PRP) Vs Hyaluronic acid (HA) for the outcomes of WOMAC stiffness at 1, 3, 6 and 12 months


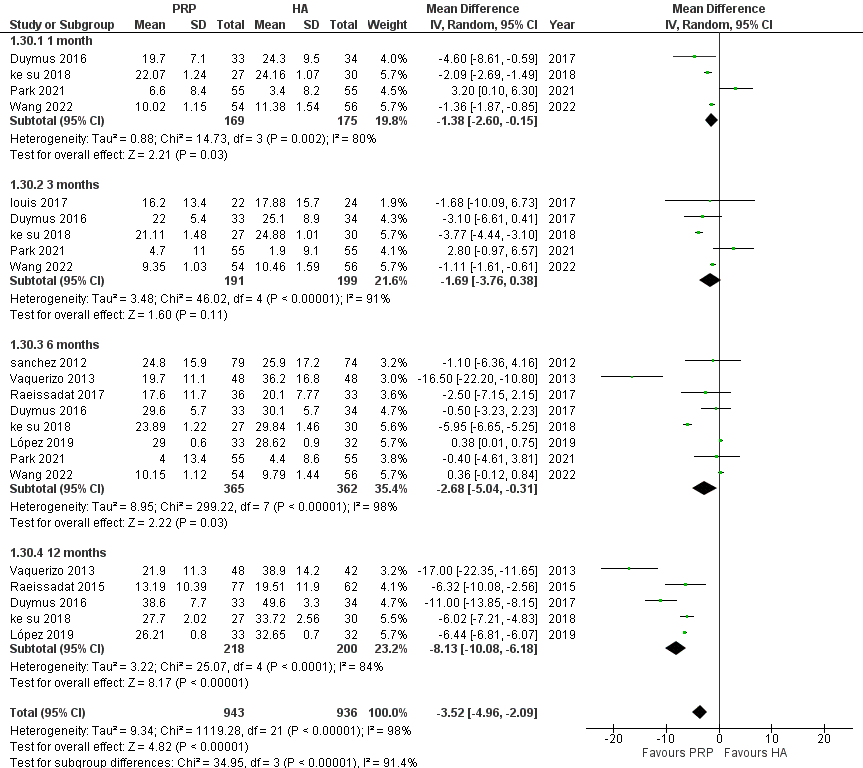


**Figure S8**: Forest plots for the subgroup analysis of Platelet rich plasma (PRP) Vs Hyaluronic acid (HA) for the outcomes of WOMAC function at 1, 3, 6 and 12 months

**
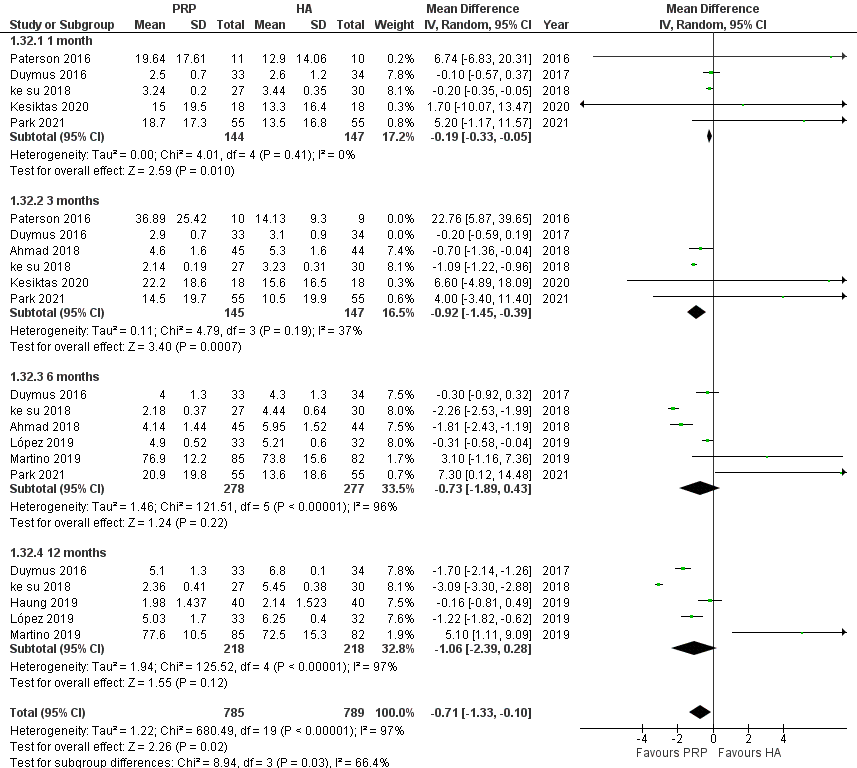
**

**Figure S9:** Sensitivity analysis for the subgroup Platelet rich plasma (PRP) vs Hyaluronic acid (HA) for the outcome of VAS pain at 3 months

**
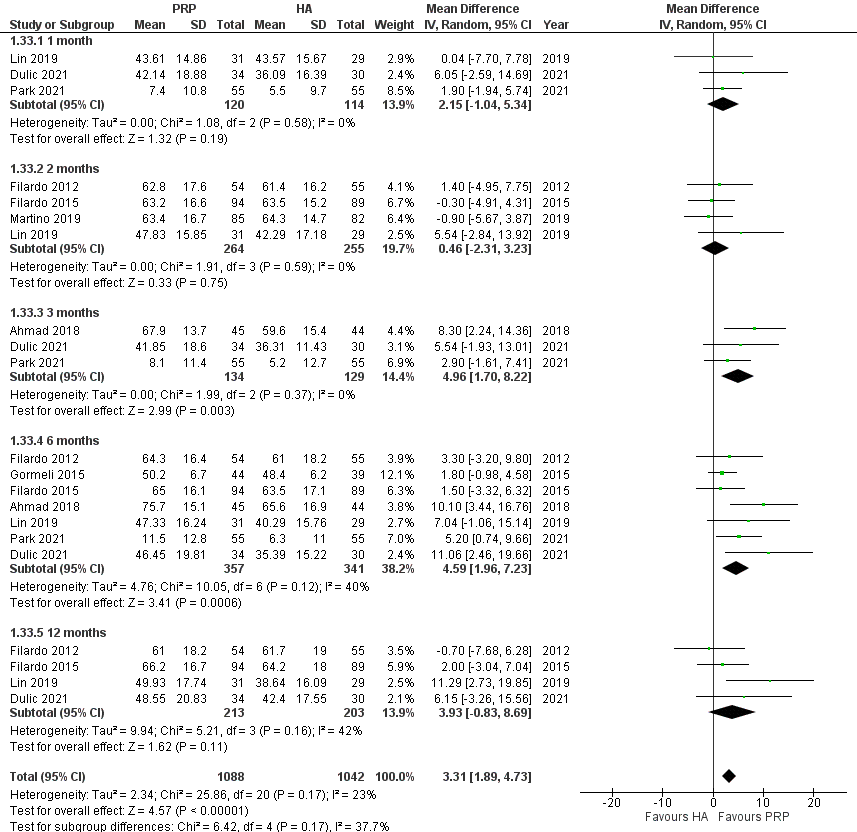
**

**Figure S10:** Forest plots for the subgroup Platelet rich plasma (PRP) Vs Hyaluronic acid (HA) for the outcomes of IKDC at 1, 2, 3, 6, and 12 months

**
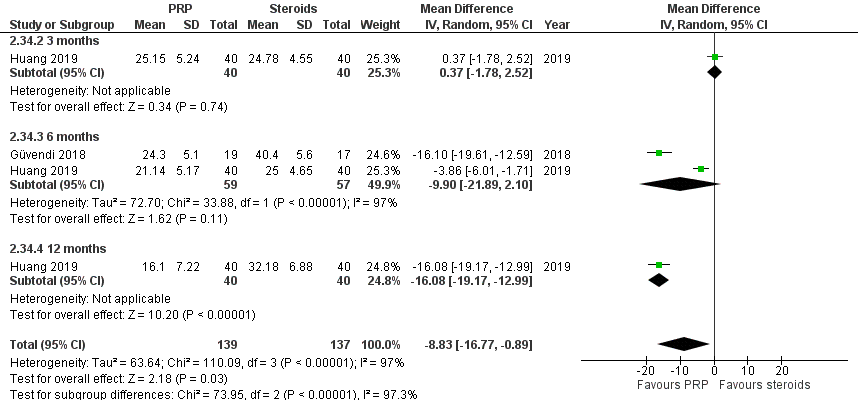
**

**Figure S11:** Forest plots for the subgroup analysis of Platelet rich plasma (PRP) Vs Steroids for the outcomes of WOMAC total at 3, 6, and 12 months

**
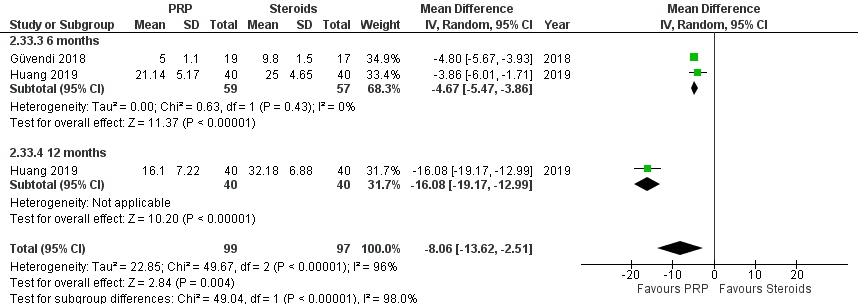
**

**Figure S12**: Forest plots for the subgroup analysis of Platelet rich plasma (PRP) Vs Steroids for the outcomes of WOMAC pain at 6 and 12 months


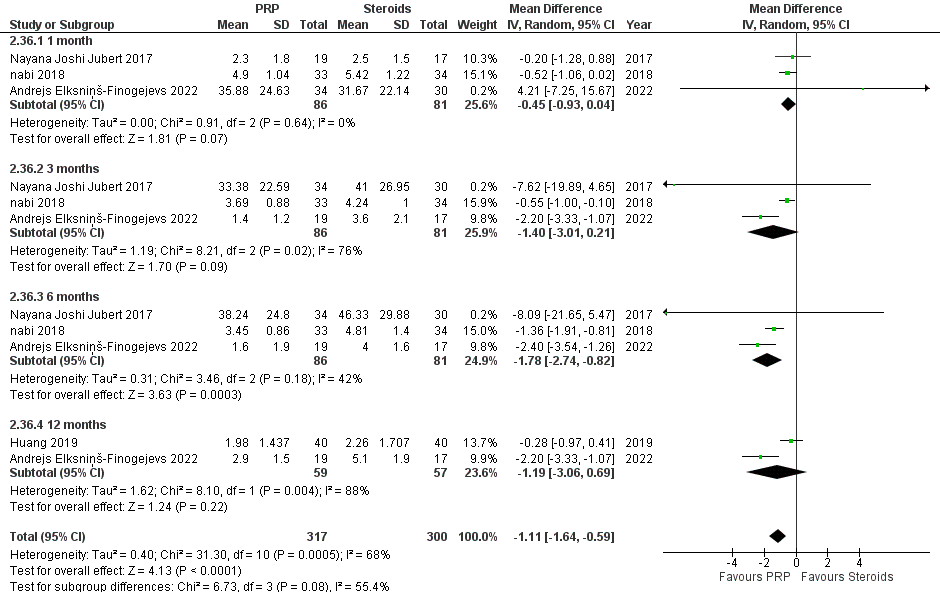


**Figure S13:** Forest plot for the subgroup analysis of Platelet rich plasma (PRP) Vs Steroids for the outcomes of VAS pain at 1, 3, 6, and 12 months


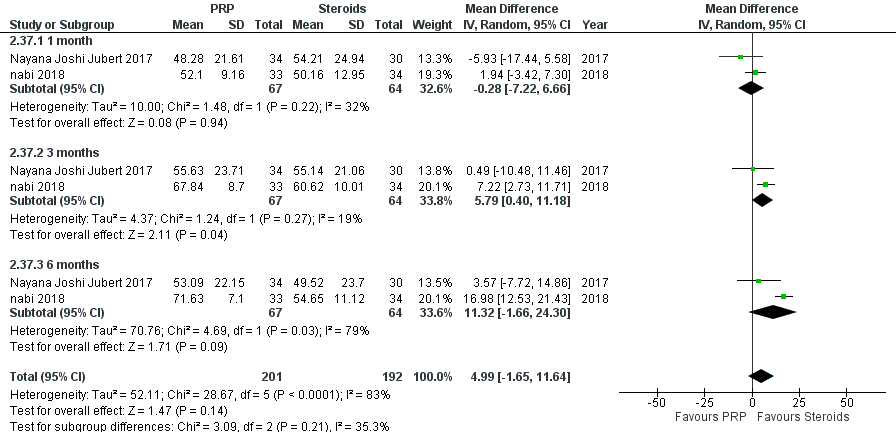


**Figure S14:** Forest plots for the subgroup analysis of Platelet rich plasma (PRP) Vs Steroids for the outcomes of KOOS pain at 1, 3, and 6 months


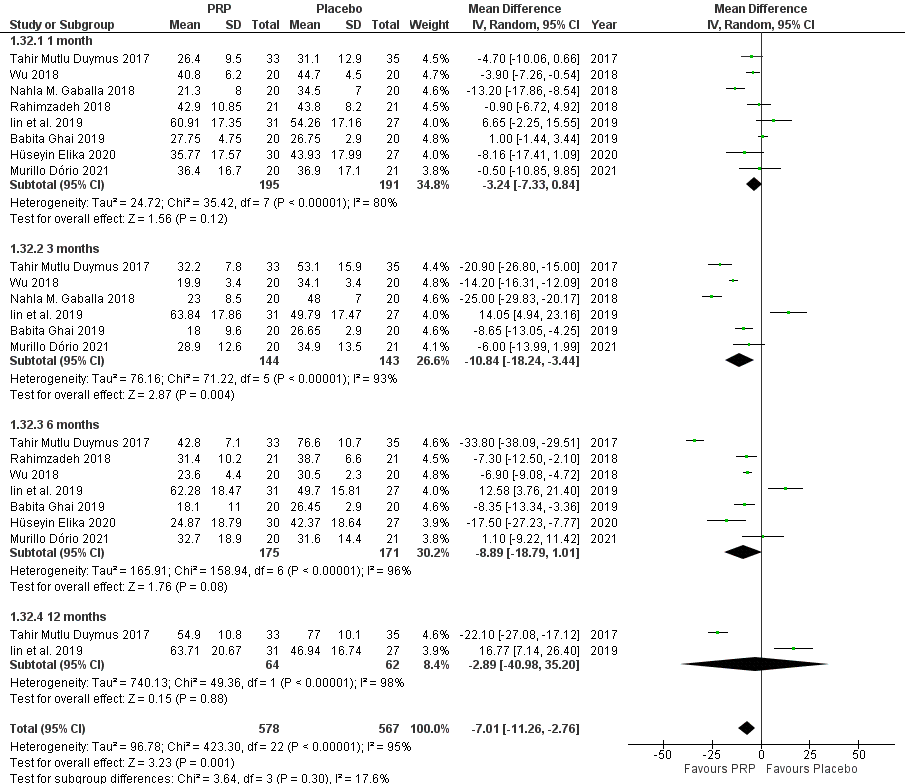


**Figure S15:** Forest plots for the subgroup analysis of Platelet rich plasma (PRP) Vs Placebo for the outcomes of WOMAC total at 1, 3, 6 and 12 months

**
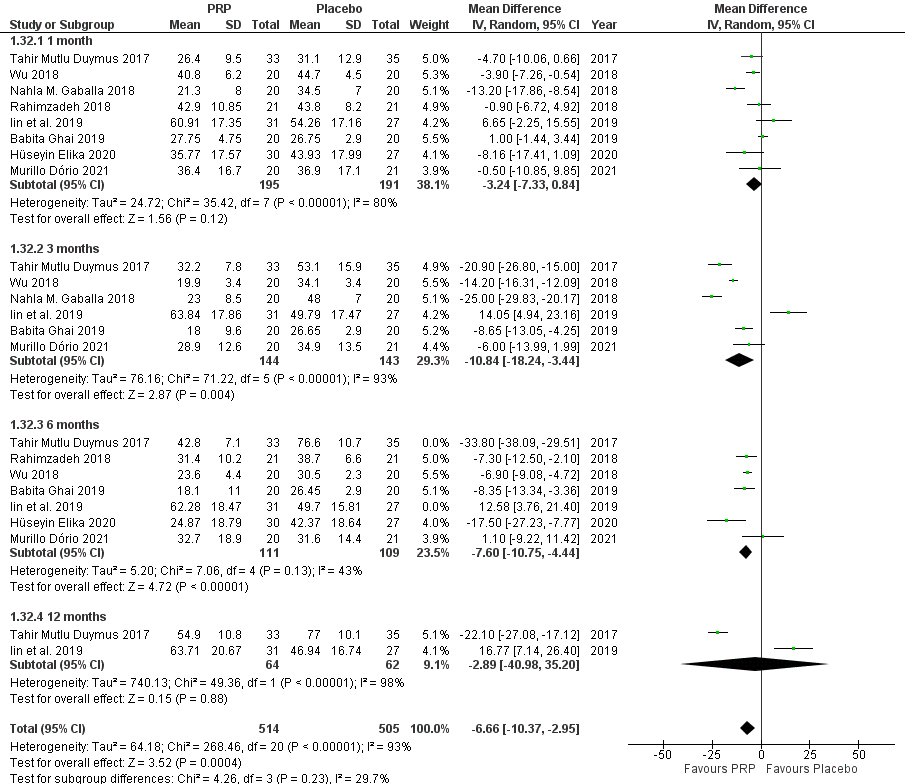
**

**Figure S16:** Sensitivity analysis for the subgroup Platelet rich plasma (PRP) vs placebo for the outcomes of WOMAC total at 6 month

**
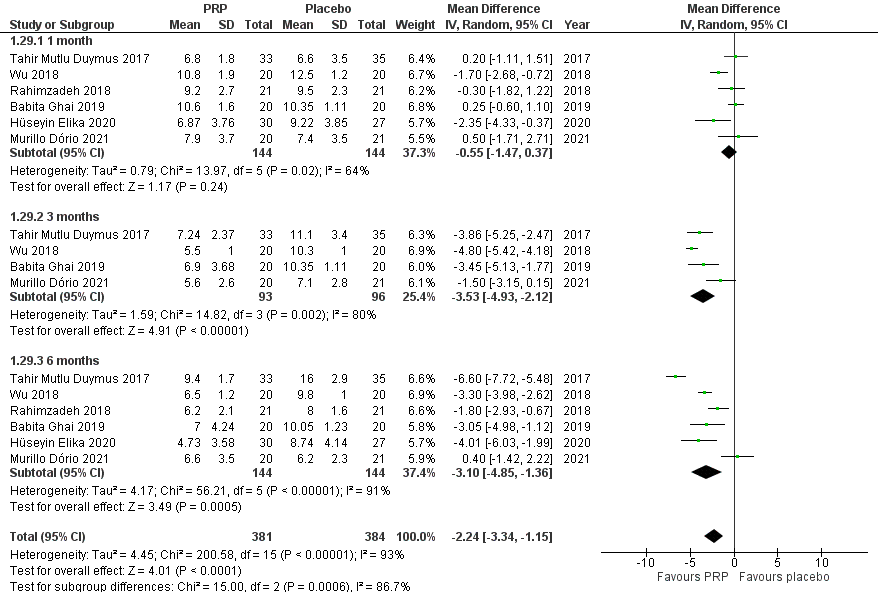
**

**Figure S17:** Forest plots for the subgroup analysis of Platelet rich plasma (PRP) Vs Placebo for the outcomes of WOMAC pain at 1, 3 and 6 months


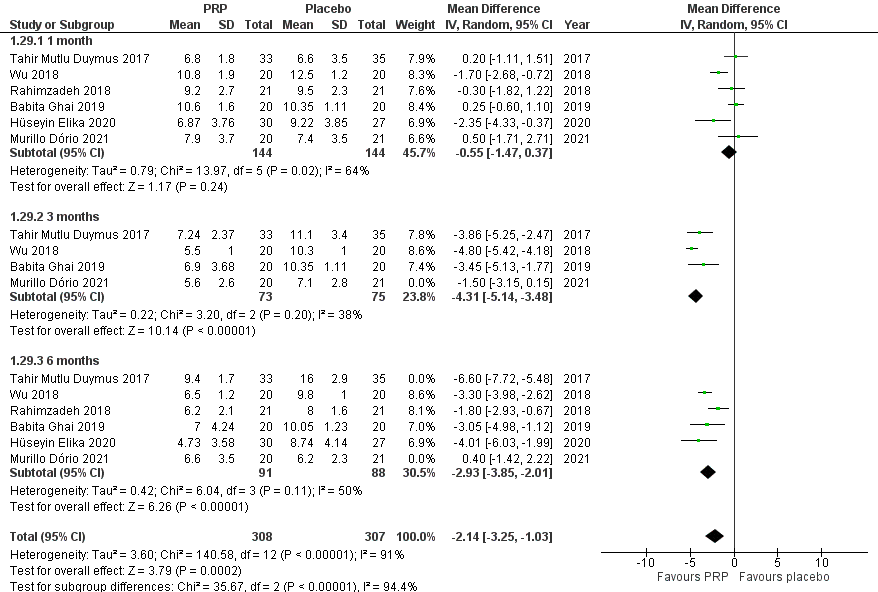


**Figure S18:** Sensitivity analysis for the subgroup Platelet rich plasma (PRP) vs placebo for the outcomes of WOMAC pain at 3 months and 6 months


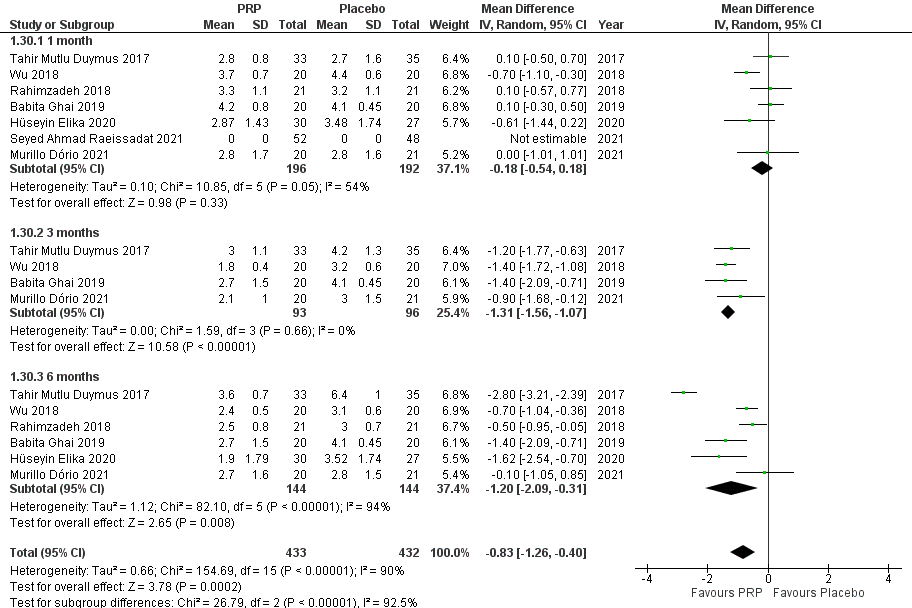


**Figure S19:** Forest plots for the subgroup analysis of Platelet rich plasma (PRP) Vs Placebo for the outcomes of WOMAC stiffness at 1, 3, and 6 months

**
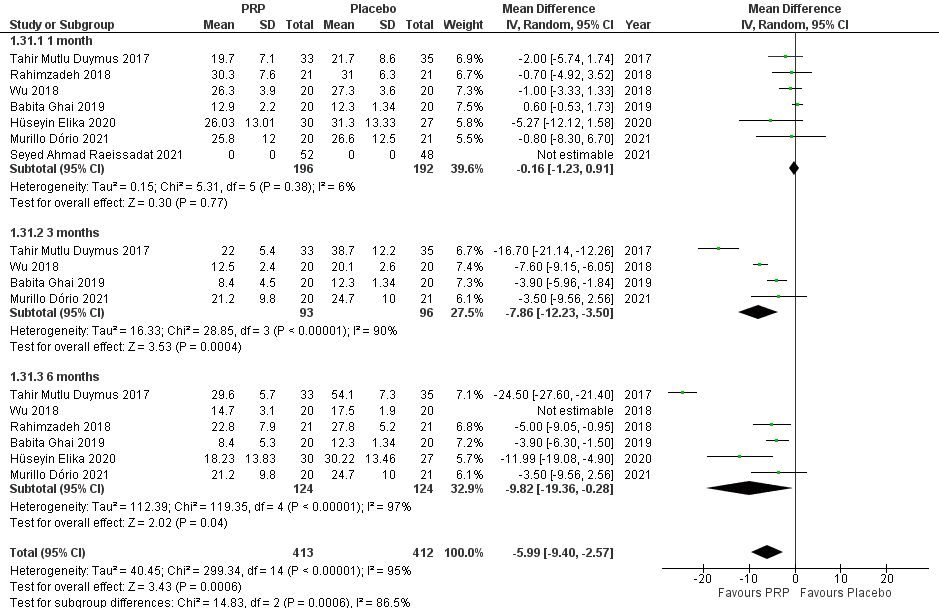
**

**Figure S20:** Forest plots for the subgroup analysis of of Platelet rich plasma (PRP) Vs Placebo for the outcome of WOMAC function at 1, 3 and 6 months

**
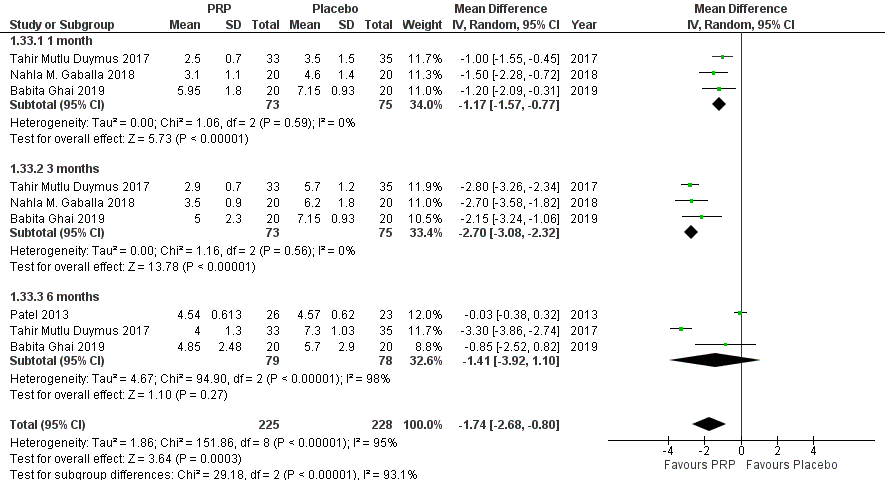
**

**Figure S21:** Forest plots for the subgroup analysis of Platelet rich plasma (PRP) Vs Placebo for the outcomes of VAS pain at 1, 3, and 6 months
